# Spatio-temporal epidemiology and associated indicators of COVID-19 (wave-I and II) in India

**DOI:** 10.1038/s41598-023-50363-2

**Published:** 2024-01-02

**Authors:** Karuppusamy Balasubramani, Venkatesh Ravichandran, Kumar Arun Prasad, Mu. Ramkumar, Sulochana Shekhar, Meenu Mariya James, Naveen Kumar Kodali, Sujit Kumar Behera, Natarajan Gopalan, Rakesh Kumar Sharma, Devojit Kumar Sarma, M. Santosh, Aditya Prasad Dash, Praveen Balabaskaran Nina

**Affiliations:** 1 Department of Geography, School of Earth Sciences, Central University of Tamil Nadu, Thiruvarur, 610005 India; 2 Department of Civil Engineering, Indian Institute of Technology Guwahati, Guwahati, 781039 India; 3 Department of Geology, Periyar University, Salem, India; 4 Department of Epidemiology and Public Health, School of Life Sciences, Central University of Tamil Nadu, Thiruvarur, 610005 India; 5 Shree Guru Gobind Singh Tricentenary University, Gurugram, New-Delhi-NCR 122505 India; 6 ICMR- National Institute for Research in Environmental Health, Bhopal Bypass Road, Bhouri, Bhopal, Madhya Pradesh India; 7 School of Earth Sciences and Resources, China University of Geosciences, Beijing, People’s Republic of China; 8 Department of Earth Sciences, University of Adelaide, Adelaide, SA Australia; 9 Asian Institute of Public Health University, Phulnakhara, Cuttack, Odisha 754001 India; 10 Department of Public Health and Community Medicine, Central University of Kerala, Kasaragod, Kerala 671316 India

**Keywords:** Public health, Epidemiology, Infectious diseases, Risk factors

## Abstract

The spatio-temporal distribution of COVID-19 across India’s states and union territories is not uniform, and the reasons for the heterogeneous spread are unclear. Identifying the space–time trends and underlying indicators influencing COVID-19 epidemiology at micro-administrative units (districts) will help guide public health strategies. The district-wise daily COVID-19 data of cases and deaths from February 2020 to August 2021 (COVID-19 waves-I and II) for the entire country were downloaded and curated from public databases. The COVID-19 data normalized with the projected population (2020) and used for space–time trend analysis shows the states/districts in southern India are the worst hit. Coastal districts and districts adjoining large urban regions of Mumbai, Chennai, Bengaluru, Goa, and New Delhi experienced > 50,001 cases per million population. Negative binomial regression analysis with 21 independent variables (identified through multicollinearity analysis, with VIF < 10) covering demography, socio-economic status, environment, and health was carried out for wave-I, wave-II, and total (wave-I and wave-II) cases and deaths. It shows wealth index, derived from household amenities datasets, has a high positive risk ratio (RR) with COVID-19 cases (RR: 3.577; 95% CI: 2.062–6.205) and deaths (RR: 2.477; 95% CI: 1.361–4.506) across the districts. Furthermore, socio-economic factors such as literacy rate, health services, other workers’ rate, alcohol use in men, tobacco use in women, overweight/obese women, and rainfall have a positive RR and are significantly associated with COVID-19 cases/deaths at the district level. These positively associated variables are highly interconnected in COVID-19 hotspot districts. Among these, the wealth index, literacy rate, and health services, the key indices of socio-economic development within a state, are some of the significant indicators associated with COVID-19 epidemiology in India. The identification of district-level space–time trends and indicators associated with COVID-19 would help policymakers devise strategies and guidelines during public health emergencies.

## Introduction

The ongoing coronavirus disease 2019 (COVID-19) pandemic that started in December 2019 has devastated millions of families by affecting physical, mental and economic health. India’s first COVID-19 wave peaked in September 2020, and the cases gradually declined until February 2021. The first COVID-19 wave in India was relatively mild, especially due to the nationwide lockdown. However, the second COVID-19 wave (February–December, 2021) was devastating, with widespread mortality and morbidity. During the course of the second wave, the Delta variant (B.1.1617.2) of SARS-CoV-2, initially identified in the state of Maharashtra in late 2020, outcompeted the pre-existing variants Kappa (B.117.1), and Alpha (B.1.1.7), and became the dominant variant^1^. The third COVID-19 wave in India started in late December 2021 and has been driven by the Omicron variant (B.1.1.529). India is the most affected country, second only to the USA in the number of cases, and third behind the USA and Brazil in deaths^2^. As of July 2022, India has witnessed > 42 million cases and > 0.5 million deaths due to COVID-19. The states of Maharashtra and Kerala rank number one and two in the total cumulative number of COVID-19 cases (7,800,000 and 6,500,000, respectively) and deaths (150,000 and 70,000, respectively)^3^.

Socio-economic factors, race, human behavior, pollution levels, exposure density, urban–rural differences, access to medical facilities, pre-existing non-communicable disease conditions, and environmental factors are some of the major influential COVID-19 explanatory variables identified worldwide^4^. Among the 28 states and eight union territories (UT) in India, there are significant geographical differences that influence COVID-19 epidemiology. Literacy levels, state’s economy, urban percent, 15–59 age group population, population density, the testing ratio at the district level, non-communicable diseases (NCDs), nutritional status, meteorological variables, and PM_2.5_ have been found to be positively associated with COVID-19 burden in India^5–8^. These studies have used partial state/district-wise COVID-19 datasets prior to the completion of the second wave and have not included all the possible explanatory variables that could affect COVID-19. Furthermore, little is known about the effect of the wealth index, current NCD rates, and worker population rates on COVID-19 cases and deaths at the district level across India.

Given the background, this study is aimed to spatio-temporally map the spread of COVID-19 in wave-I and wave-II at the district level across India for the identification of COVID-19 hotspots. Furthermore, another major objective is to identify the environmental, demographic, socio-economic, and health factors associated with district-level COVID-19 rates to help guide public health strategies.

## Methods

### Study design

This is a cross-sectional study that compares COVID-19 cases/deaths and their associated indicators at different geographical locations in India at a defined time interval, with individual districts as the unit of measurement. The data on COVID-19 cases and deaths over the course of COVID-19 (waves-I and II) and important geographical variables at the district level were used for spatial and statistical analysis.

### Spatial data preparation

Even though the COVID-19 wave-II in India lasted till December 2021, only the period from February 2020 to August 2021 (wave-I and wave-II) was considered because the publicly available COVID-19-related datasets from https://data.covid19india.org/ and https://www.mohfw.gov.in/ were not regularly updated since September 2021. The datasets were further segregated based on wave-I (April 2020 to January 2021) and wave-II (February 2021 to August 2021). The administrative unit (i.e., district) of the 2011 Census of India was considered for uniform data assimilation. The data of newer districts (demarcated after the 2011 Census) were combined with their parent district. The districts of megacities such as Mumbai and New Delhi were clubbed into a single unit to avoid inconsistencies in comparing COVID-19 cases/deaths with other variables. The COVID-19 incidence and mortality data were normalized according to the projected population (2020)^9^ and used for space–time trend analysis.

Given India’s diversified geography and socio-economic status^10,11^, 28 independent variables related to the environment, demography, socio-economic, and health aspects were selected to study their association with COVID-19 cases and deaths. The source of the environmental, demographic, socio-economic, and health datasets are listed in Table 1. The 2011 census data (the latest available) was downloaded from the Census of India portal to compute demographic, household, wealth, literacy, occupation, and infrastructure characteristics at the district level. The key health variables, such as anemia, blood sugar level, blood pressure, tobacco and alcohol consumption, and obesity were obtained from the National Family Health Survey-5 (NFHS-5), carried out from 2019 to 2021.

**Table 1:** Dependent and independent variables used for spatial and statistical analysis, and their data sources.

Category	Variable	Description of the variable	Period	Source
COVID-19 (dependent variables)**	Wave-I cases	Wave-I cases (April 2020 to January 2021)	2020–2021	https://data.covid19india.org/ https://www.mohfw.gov.in/
	Wave-I deaths	Wave-I deaths (April 2020 to January 2021)		
	Wave-II cases	Wave-II cases (February 2021 to August 2021)		
	Wave-II deaths	Wave-II deaths (February 2021 to August 2021)		
	Total cases	Total cases recorded (February 2020 to August 2021)		
	Total deaths	Total deaths recorded (February 2020 to August 2021)		
Demographic and socio-economic	Population	Projected population	2020	https://dataverse.harvard.edu/dataset.xhtml?persistentId=doi:10.7910/DVN/RXYJR6
	Population-2011	Census population	2011	https://censusindia.gov.in/census.website
	Household density	No. of households/sq. km area		
	Population density	No. of persons/sq. km area		
	SC/ST population	Scheduled Castes/Tribes population density (no. of persons/sq. km area)		
	Literacy	Literacy rate (%)		
	Working population	Total working population rate (%)		
	Cultivators	Cultivator population rate (%)		
	Agricultural laborers	Agricultural laborers’ rate (%)		
	Household industries workers	Household industries workers’ rate (%)		
	Other workers	Other workers’ rate (%)		
	Wealth index	Wealth index (0–1) is computed based on household indicators (Refer Supplementary File 1)		
	Health services	No. of beds in all kind of hospitals		
Environmental	Forest	Forest cover (%)	2019	https://fsi.nic.in/forest-report-2019
	Elevation	Average elevation (meters above mean sea level)	2007	https://developers.google.com/earth-engine/datasets/catalog/USGS_SRTMGL1_003
	Max temp	Annual average maximum temperature (℃)	2018–2021	https://developers.google.com/earth-engine/datasets/catalog/IDAHO_EPSCOR_TERRACLIMATE
	Min temp	Annual average minimum temperature (℃)		
	Windspeed	Annual average windspeed (km/h)		
	AET	Annual average actual evapotranspiration (mm)		
	Rainfall	Annual average rainfall (mm)		https://chrsdata.eng.uci.edu/
	PM_2.5_	Annual average particulate matter 2.5 (μg/m^3^)		https://sites.wustl.edu/acag/datasets/surface-pm2-5/
Health	High blood glucose	Blood sugar level > 140 mg/dl or taking medicine to control blood sugar level (%)	2019–2021	http://rchiips.org/nfhs/
	High blood pressure	Elevated blood pressure (systolic ≥ 140 mmHg and/or diastolic ≥ 90 mmHg) or taking medicine to control blood pressure (%)		
	Tobacco (women)	Women age 15 years and above who use any kind of tobacco (%)		
	Tobacco (men)	Men age 15 years and above who use any kind of tobacco (%)		
	Alcohol (women)	Women age 15 years and above who consume alcohol (%)		
	Alcohol (men)	Men age 15 years and above who consume alcohol (%)		
	Obese (women)	Women who are overweight or obese (BMI ≥ 25.0 kg/m^2^) (%)		
	Waist-to-hip ratio	Women who have high-risk waist-to-hip ratio (≥ 0.85) (%)		
	Anemia women	Women age 15–49 years who are anemic (%)		

**Since the publicly available COVID-19 related domains such as https://data.covid19india.org/ and https://www.mohfw.gov.in/ were not regularly updated since September 2021, the period from February 2020 to August 2021 was considered.

The TERRACLIMATE datasets with ~ 4 km and ASTER datasets with 30-m spatial resolutions^12^ were used to extract elevation and climatic parameters (except rainfall) using the Google Earth Engine (GEE) platform. The PERSIANN-Cloud Classification System (PERSIANN-CCS) datasets (https://chrsdata.eng.uci.edu/) and Forest Survey of India (FSI) 2019 annual reports were used respectively for rainfall^13^ and forest data^14^. The particulate matter 2.5 (PM_2.5_) concentration was obtained from the combined measurement of satellite (NASA MODIS, MISR, and SeaWiFS) derived Aerosol Optical Depth (AOD) and ground-derived PM_2.5_ datasets^15^. All these environmental datasets were aggregated at the district level to extract a mean value for each district. In order to remove the seasonal effects and short-term changes, an annual average value of 2018–2021 is considered for the climatic datasets.

As there are no readily available datasets at the district level for assessing wealth and health services, these two variables were determined based on the parameters extracted from the district census handbooks^10^. The health services variable was determined based on the availability of all kinds of hospital beds in a district. The methodology for deriving the wealth index is provided in Supplementary File 1.

### Space–time trend analysis

The emerging hotspot analysis is a geospatial technique used to understand space–time dynamics through the identification of hot and coldspots of any spatial event^16^. The COVID-19 datasets were prepared as population (2020) normalized 15 days sum of cases and deaths (Supplementary Files 2, 3). The data were then converted into a space–time cube using ArcGIS Pro software (version 2.8.0; year 2021), and a total of 34-time steps with 21,760 observations/bins (640 spatial units multiplied by 34 temporal intervals) were generated, and each bin was considered as an observation. The output was distributed into hotspots (highest significant value over the region) and coldspots (lowest significant value over the region)^17^. Theoretically, the emerging hotspot analysis was computed through the Getis-Ord Gi* formula given in Eqs. (1) and (2).

1 $$G_{i}^{*} = \frac{{\sum\nolimits_{j}^{n} {w_{i,j} \,x_{j} - \overline{X} \sum\nolimits_{j}^{n} {w_{i,j} } } }}{{\sqrt[S]{{\frac{{\left[ {n\sum\nolimits_{j - 1}^{n} {w_{{_{i,j} }}^{2} } - \left( {\sum\nolimits_{j - 1}^{n} {w_{i,j} } } \right)^{2} } \right]}}{n - 1}}}}},$$

2 $$\overline{X} = \frac{{\sum\nolimits_{j = 1}^{n} {x_{j} } }}{n},\,\,S = \sqrt {\frac{{\sum\nolimits_{j = 1}^{n} {x_{{_{j} }}^{2} } }}{n}} - (\overline{X} )^{2} ,$$

where *x*_*j*_ is the attribute value for spatial feature *j*, *w*_*i,j*_ is the spatial weight between features *i* and *j*, and *n* is the total number of features^18,19^.

At each iteration, a space–time bin was generated (containing z-score and p-value) and used to determine the hotspots using the Mann–Kendall trend test^17,20^. The metadata of space–time analysis is provided in Supplementary File 4. The hotspot bins were classified into six classes: consecutive (uninterrupted run of hot time step intervals), intensifying (at least 90% of the bins becoming hotter over time), persistent (at least 90% of the bins are hot but with no trend up or down), diminishing (at least 90% of the bins becoming less hot over time), sporadic (some of the time step intervals are hot) and oscillating (some of the time step intervals are hot, some are cold)^21^.

### Negative binomial regression analysis

The linear regression model is frequently applied to count variables under the assumption that they are continuous normally distributed, but this can lead to inaccurate, inconsistent, and biased results. The Poisson Regression Model (PRM) specifically addresses count outcomes; the probability distribution of a count in the PRM is defined by a Poisson process, where the distribution’s mean is a function of the independent variables. The variance and mean of the outcome are assumed equal (equal dispersion), but most often, the variance exceeds the mean (over-dispersion). To overcome this issue, the Negative Binomial Regression Model (NBRM) is used, which allows the variance to be greater than the mean^22,23^ and hence considered the best-fitting model for count data^24^. In this case too, the six dependent variables (COVID-19 cases/deaths: Total, wave-I, and wave-II) are non-negative count data, and the variance is greater than the mean for each dependent variable. Hence, the NBRM statistical test is used to adjust for over-dispersion in the outcome variables and to assess the association between the geographic variables and COVID-19 cases and deaths (wave-I, wave-II, and total) in India at the district level from February 2020 to August 2021 (Supplementary File 5). Based on exploratory regression (spatial) analysis, all overlapping variables were removed through the iterative steps, and 21 independent variables from demographic (household density, literacy rate), socio-economic (agricultural laborers’ rate, household industries workers’ rate, other workers’ rate, wealth index, and health services), environment (forest, minimum temperature, wind speed, actual evapotranspiration, rainfall, and PM_2.5_), and health (high blood glucose, high blood pressure, tobacco women, tobacco men, alcohol women, alcohol men, anemia women, and overweight/obese women) were used for the regression models as listed in Supplementary File 6. All the independent variables were assessed with the cases and deaths for each of the waves (wave-I, wave-II, and total), with the 2020 projected population of each district as an offset variable. The independent variables with P < 0.2 identified through univariable analyses were used to run the multivariable analyses (full model). To obtain a reduced model through iterative multivariable fitting, the variables were removed one at a time as they were not significant in the multivariable model at the alpha level of 0.10 and, when taken out, did not change any of the remaining parameter estimates by > 20%. The likelihood ratio test (Supplementary File 7) shows the reduced model to be as good as the full model in fitting the data; the reduced model was chosen for the principle of parsimony (Supplementary File 7). To ensure linearity, a few independent variables were transformed (log of household density, square root of agriculture, square root of alcohol women, log of forest, and square root of health services), and used for the analysis. Multicollinearity was assessed between independent variables, and variables with variance inflation factor (VIF) greater than 10 were eliminated. The risk ratios for COVID-19 cases (wave-I, wave-II, and total) and deaths (wave-I, wave-II, and total) were determined separately using six regression models. Each model’s risk ratio (RR) value is reported with its 95% confidence interval and P-value. The results of each model, along with residual plots for reduced models, are presented in Supplementary File 7. The NBRM statistical analysis and exploratory regression (spatial) analysis were carried out in STATA software (Version 17, year 2021) and ArcGIS Pro software (2.8.0), respectively.

### Spatial mapping

The district-wise spatio-temporal trends in COVID-19 cases and deaths were mapped using choropleth mapping techniques. Among the 21 variables used for statistical analysis, only nine (wealth index, health services, literacy rate, household density, other workers’ rate, blood sugar level, forest cover, annual rainfall, and PM_2.5_) were mapped based on the results from negative binomial regression analysis and spatial exploratory regression analysis of COVID-19 cases/deaths. All the maps were created using ArcGIS 10.4 software (year 2016) with online base map services^25^.

## Results

### Spatio-temporal pattern of COVID-19 cases and deaths

**Figure 1:**
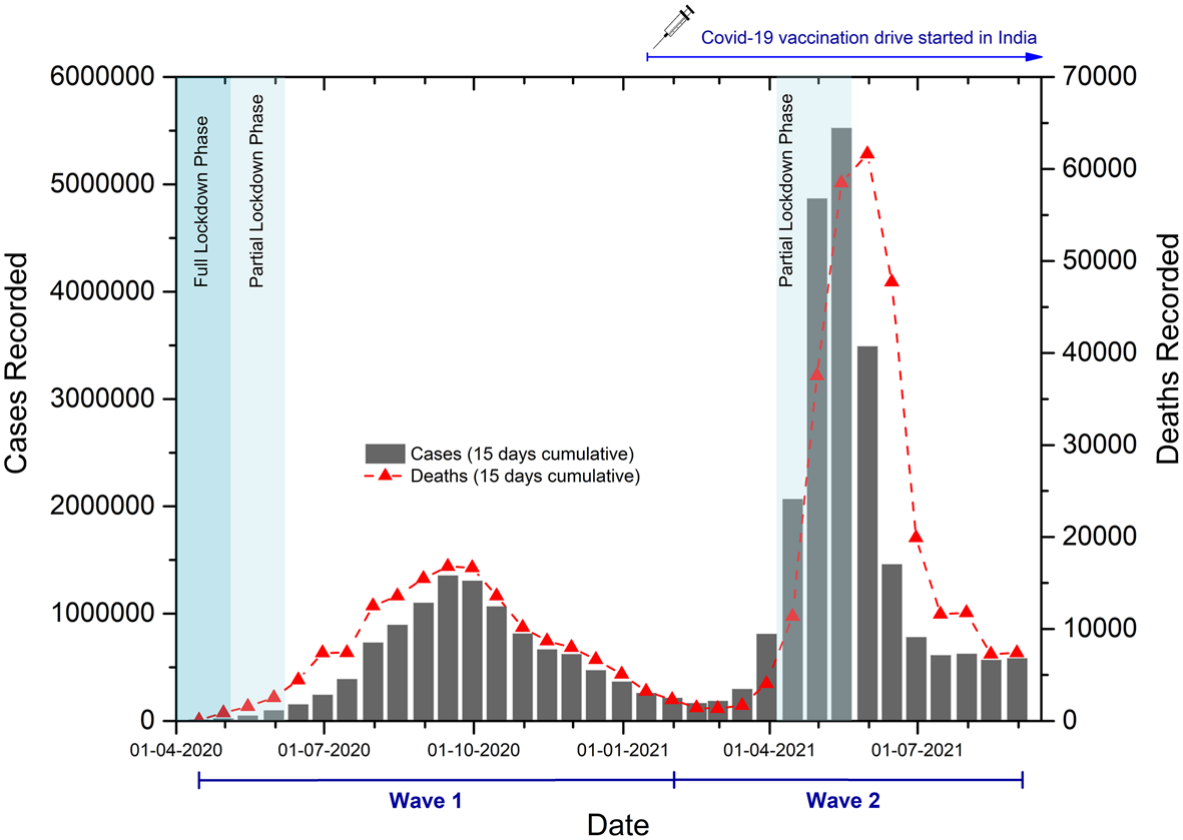
The cumulative COVID-19 cases and deaths in India from April 2020 to August 2021. The timeline for waves I and II is represented on the x-axis. The major lockdown periods are shaded and labeled.

The district-wise data of COVID-19 cases and deaths, from February 2020 to August 2021, were aggregated and used to generate the 15 days cumulative sum for the entire country. As 15 days cumulative cases and deaths were negligible until 31^st^ March 2020, data from 01st April 2020 to 31st August 2021 were analyzed as shown in Fig. 1. During the first wave, the maximum number of cases (1,356,052) and deaths (16,787) were recorded between 01st and 15th September 2020. From then, the cases started decreasing gradually and were at their lowest in February 2021. In the second wave, the cases and deaths reached the peak in the second week of May 2021 (5,522,829 cases and 61,625 deaths were recorded between 01st and 15th May 2021).

**Figure 2:**
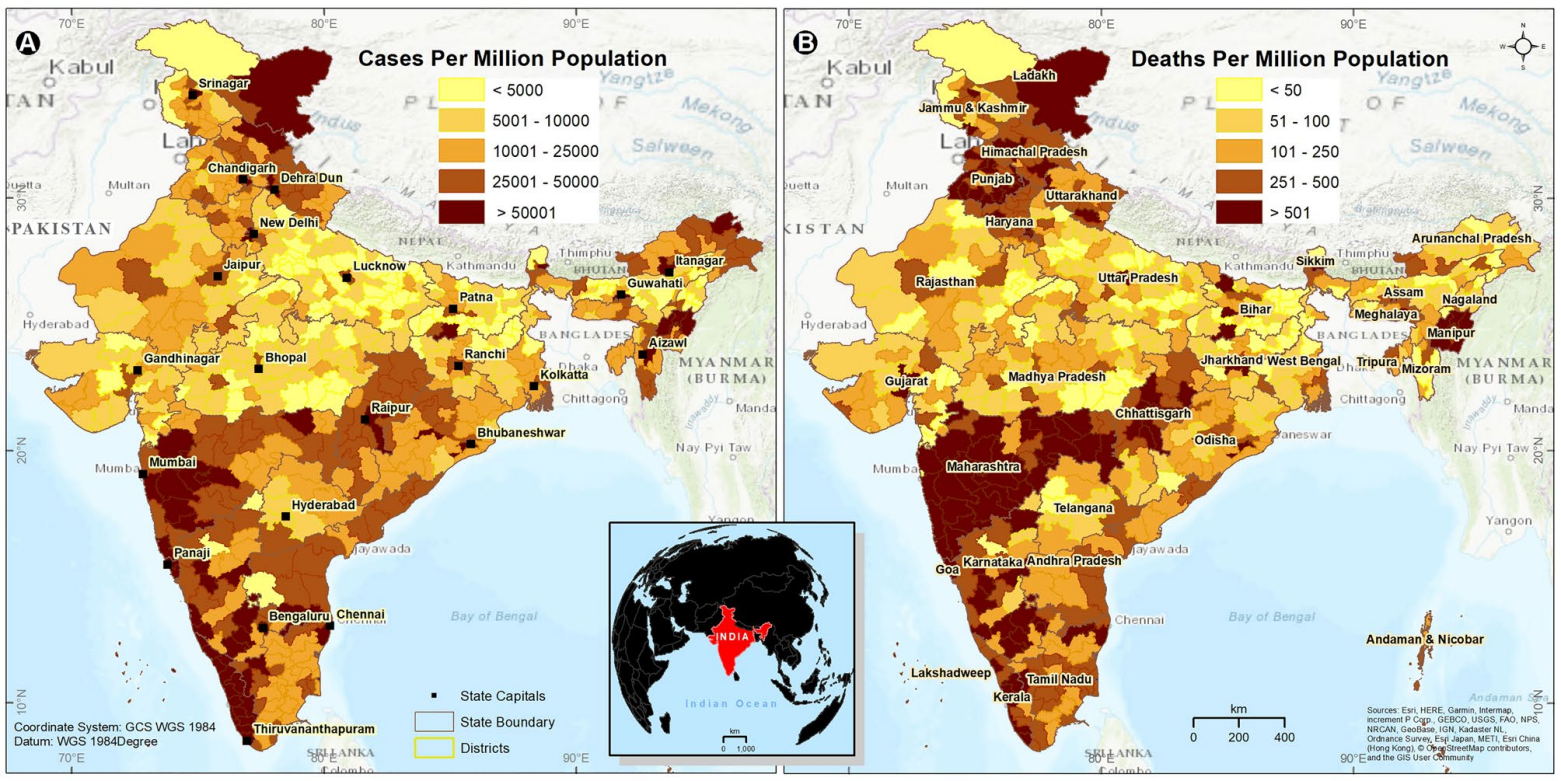
District-wise (**A**) COVID-19 cases per million population and (**B**) deaths per million population in India from April 2020 to August 2021. The inset map shows the location of India in Asia. The map was created using the licensed version of ArcGIS 10.4 software by Esri (www.esri.com). The background map represents the World Topographic Map (http://goto.arcgisonline.com/maps/World_Topo_Map).

The spatial distributions of the district-wise COVID-19 cases and deaths normalized per million population for wave-I and wave-II are shown in Fig. 2, and the cumulative sums for the same periods are presented in Supplementary Fig. 8 (S8.1, S8.2, S8.3, S8.4). The state of Kerala is the worst hit, and is closely followed by the neighboring urban districts of Mumbai in Maharashtra. Large urban regions, such as Chennai, Bengaluru, Goa, New Delhi, and their adjoining districts, also reported very high caseloads (> 50,001 per million). Also, the urban districts in the North-Eastern (NE) states of Assam, Sikkim, Manipur, Mizoram, and Arunachal Pradesh reported high number of cases. Interestingly, the cases per million population are high even in the less densely populated districts of Ladakh and Dibang Valley in Arunachal Pradesh, and Lahaul and Spiti in Himachal Pradesh. On the other hand, the caseload was less (< 5000 per million) in many districts of Uttar Pradesh, Madhya Pradesh, and Bihar, states with high population density.

The spatial distribution of the deaths (> 501 per million) shows the state of Maharashtra to be the worst affected. The densely populated urban districts in the states of Kerala, Karnataka, Tamil Nadu, Chhattisgarh, Odisha, Sikkim, Uttarakhand, Jammu and Kashmir, Himachal Pradesh, Bihar, Punjab, Haryana, Uttar Pradesh, and Gujarat also reported > 501 deaths per million population. Among the NE states, Manipur reported > 501 deaths per million population. COVID-19 deaths were low (< 50 to 250 per million) in many districts of Rajasthan, Madhya Pradesh, Andhra Pradesh, Telangana, Odisha, West Bengal, Bihar, Gujarat, and Jharkhand.

### Spatial pattern of key geographical variables

**Figure 3:**
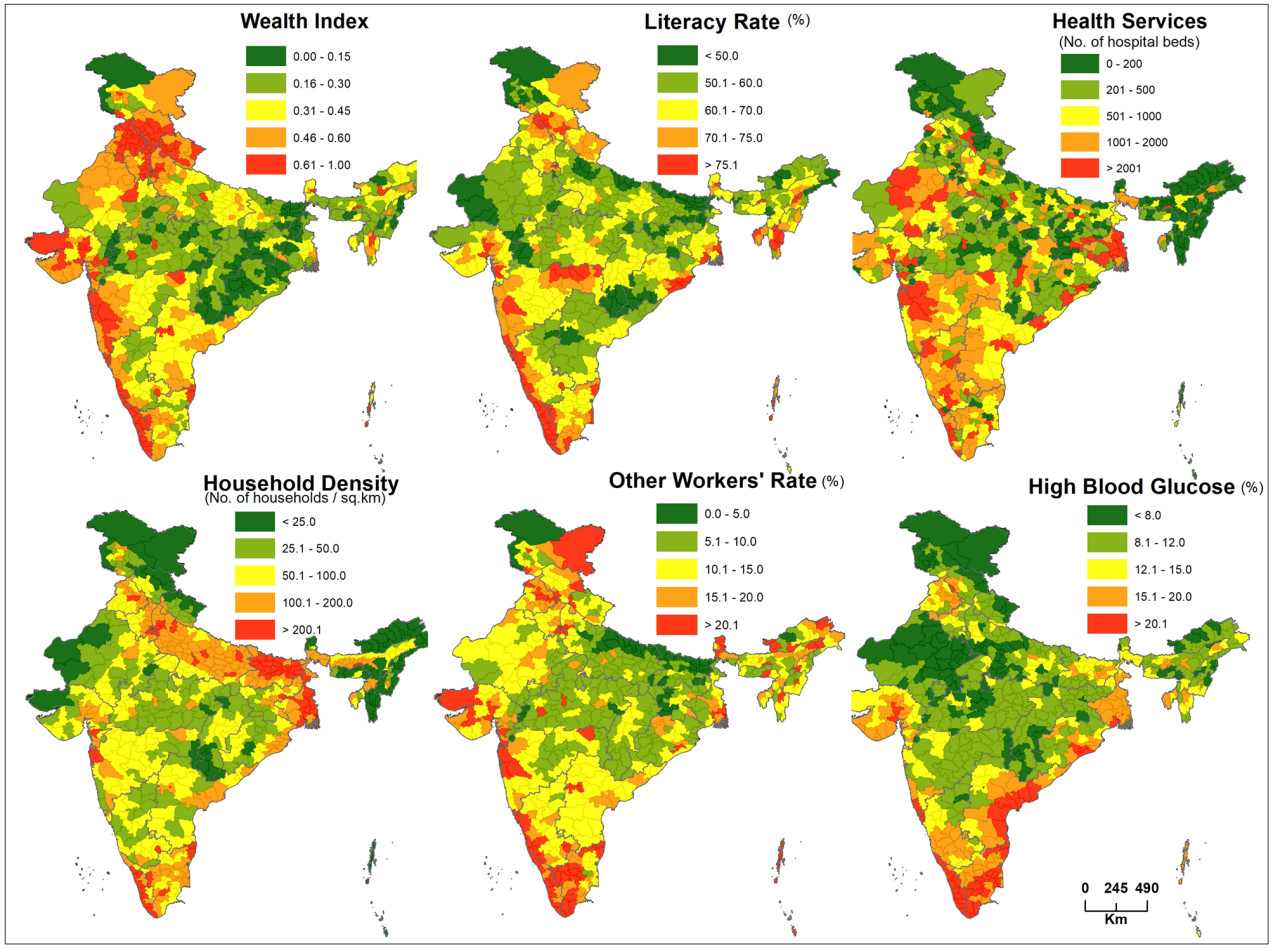
Spatial distributions of the influential socio-economic, demographic, and health indicators at the district level in India. The map was created using the licensed version of ArcGIS 10.4 software by Esri (www.esri.com).

The district-level spatial distributions of the key geographical variables: wealth index, literacy rate, health services, household density, other workers’ rate, and high blood glucose are shown in Fig. 3. A strong spatial overlap of these indicators with high COVID-19 cases and deaths is discernible in the states of Kerala and Maharashtra.

In addition, environmental factors (forest cover, annual rainfall, minimum temperature, and PM_2.5_ concentrations) mapped at the district level are shown in Supplementary Fig. 9. Forest cover, average annual rainfall, and average minimum temperature are positively correlated with COVID-19 cases, especially in Kerala and Maharashtra. The concentration of PM_2.5_ is negatively associated with COVID-19; the states of Haryana, Uttar Pradesh, and Bihar have high PM_2.5_ but fewer COVID-19 cases and deaths.

### Spatio-temporal trend of COVID-19 spread

**Figure 4:**
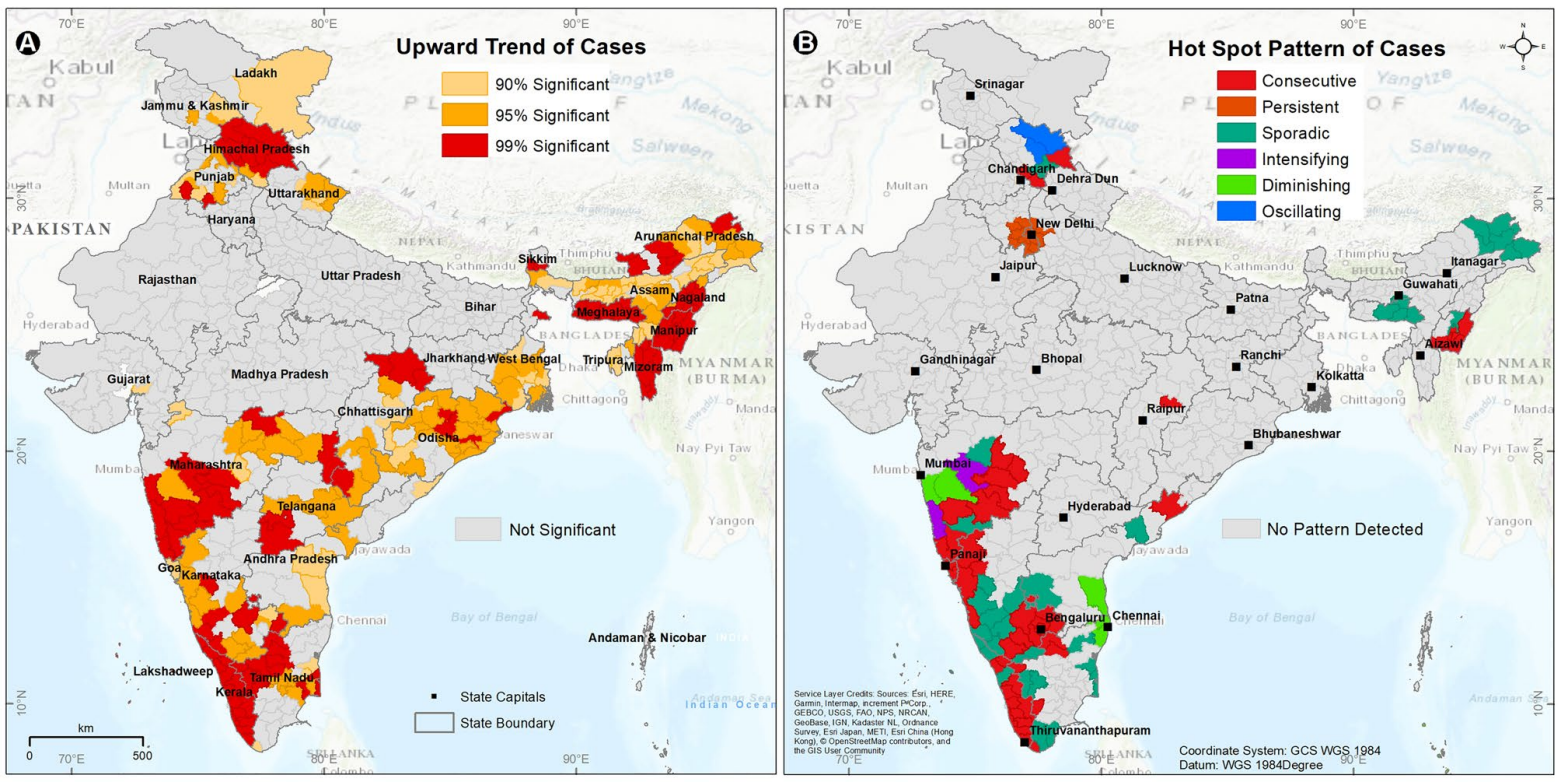
Distribution of (**A**) upward trend districts with different significance (P-value) levels and (**B**) distribution of different categories of hotspot districts identified by space–time pattern analysis for COVID-19 cases in India. The map was created using the licensed version of ArcGIS 10.4 software by Esri (www.esri.com). The background map represents the World Topographic Map (http://goto.arcgisonline.com/maps/World_Topo_Map).

The district-level space–time trends and hotspots identified by emerging hotspot analysis of population-normalized COVID-19 cases are shown in Fig. 4A,B. The trend analysis of cases over a period of 375 days (the best-fitted time period selected by the model) shows the entire state of Kerala, the western part of Tamil Nadu, districts surrounding Bengaluru, Mumbai and Hyderabad, northern districts of Chhattisgarh, most of the districts of Himachal Pradesh and NE states to have an upward trend of COVID-19 cases at 99% CI.

The entire wave’s space–time hotspot analysis of the 640 census districts finds ~ 20% of the districts (123 districts) to have different categories of hotspots. A consecutive hotspot characteristic is observed in the entire state of Goa, 11 out of the 14 districts of Kerala, 12 districts of Karnataka, 8 districts of Maharashtra, 6 districts of Manipur, 5 districts of Tamil Nadu, and 3 districts of Himachal Pradesh. The districts of Maharashtra show an intensifying hotspot characteristic, while the New Delhi region shows a persistent hotspot characteristic. Diminishing hotspot characteristic is observed in Chennai and Mumbai suburban districts. A sporadic hotspot characteristic is observed mainly in the southern and NE states, while an oscillating hotspot trend is observed in Himachal Pradesh.

### Association between the geographical variables and COVID-19

Negative binomial regression analysis results show the association between COVID-19 cases/deaths and the key demographic, socio-economic, environmental, and health-related factors, and are given in Table 2 and Supplementary Files S5, S6 and S7. Among the variables, the wealth index showed a high RR for COVID-19 cases and deaths (wave-I, wave-II, and total). The RR of COVID-19 cases increased by ~ 3 times for every unit increase in the wealth index in COVID-19 cases (total wave [RR: 3.577; 95% CI: 2.062–6.205]) and deaths (total wave [RR: 2.477; 95% CI: 1.361–4.506]). The literacy rate is the other significant variable that had a significant positive RR for COVID-19 cases (total wave [RR: 1.021; 95% CI: 1.011–1.032]) and deaths (total wave [RR: 1.040; 95% CI: 1.028–1.052]).

**Table 2:** Estimated risk ratio (RR) for factors that significantly associated with COVID-19 cases and deaths, during wave-I and wave-II, India, February 2020 to August 2021.

Variables	Cases						Deaths					
	Wave-I		Wave-II		Total wave		Wave-I		Wave-II		Total wave	
	RR	P value	RR	P value	RR	P value	RR	P value	RR	P value	RR	P value
Wealth index	3.854	0.000*	3.35	0.000*	3.577	0.000*	3.731	0.001*	2.434	0.017*	2.477	0.003*
Literacy rate (%)	–	–	1.027	0.000*	1.021	0.000*	1.027	0.000*	1.037	0.000*	1.04	0.000*
Other workers’ rate (%)	1.021	0.064	–	–	–	–	1.026	0.042*	–	–	–	–
Minimum temperature (℃)	–	–	–	–	–	–	0.998	0.871	–	–	–	–
Wind speed (km/h)	–	–	0.771	0.043*	0.779	0.046*	–	–	0.733	0.022*	0.78	0.054
Rainfall (mm)	–	–	–	–	–	–	1	0.049*	–	–	–	
PM_2.5_ (μg/m^3^)	0.991	0.000*	0.987	0.000*	0.987	0.000*	0.991	0.000*	0.99	0.003*	0.991	0.001*
Health services (%)	1.005	0.023*	1.004	0.051	1.005	0.021*	1.007	0.002*	1.005	0.025*	1.006	0.011*
Alcohol men (%)	1.014	0.000*	1.017	0.000*	1.018	0.000*	–	–	–	–	–	–
Alcohol women (%)	–	–	–	–	–	–	–	–	0.942	0.186	–	–
Tobacco men (%)	0.981	0.000*	0.99	0.017*	0.99	0.011*	0.984	0.016*	0.982	0.002*	0.974	0.000*
Tobacco women (%)	1.019	0.001*	–	–	–	–	1.017	0.006*	1.01	0.102	1.015	0.006*
High blood glucose (%)	–	–	–	–	–	–	0.933	0.000*	0.959	0.001*	0.963	0.001*
High blood pressure (%)	–	–	–	–	–	–	1.012	0.26	1.017	0.083		
Overweight/Obesity women (%)	–	–	–	–	–	–	1.02	0.007*	–	–	–	–
Anemia women (%)	1.007	0.048*	–	–	–	–	1.006	0.178	–	–	–	–

*Statistical significance at P < 0.05

“–” Variables not found significant by the model.

Other workers’ rate, health services, tobacco use in women, alcohol use in men, overweight/obesity in women, anemia in women, and rainfall were the other positively significant factors observed in the negative binomial regression analysis. With every additional unit increase in alcohol use in men (total wave [RR: 1.018; 95% CI: 1.011–1.025]), tobacco use in women (wave-I [RR: 1.019; 95% CI: 1.008–1.031]), health services (total wave [RR: 1.005; 95% CI: 1.001–1.009]), and anemia in women (wave-I [RR: 1.007; 95% CI: 1.000–1.014]), the RR of COVID-19 cases increased. Similarly, in COVID-19 deaths, with every additional unit increase in the other workers’ rate (wave-I [RR: 1.026; 95% CI: 1.001–1.051]), overweight/obesity in women (wave-I [RR: 1.020; 95% CI: 1.005–1.036]), tobacco use in women (total waves [RR: 1.015; 95% CI: 1.004–1.026]), health services (total waves [RR: 1.006; 95% CI: 1.001–1.010]), and rainfall (wave-I [RR: 1.000; 95% CI: 1.000–1.000]), the RR increased.

A negatively significant association was observed between COVID-19 cases/deaths and PM_2.5_ (total cases [RR: 0.987; 95% CI: 0.984–0.990]; total deaths [RR: 0.991; 95% CI: 0.987–0.994]), tobacco use in men (total cases [RR: 0.990; 95% CI: 0.982–0.998]; total deaths [RR: 0.974; 95% CI: 0.964–0.983]), and wind speed (total cases [RR: 0.779; 95% CI: 0.609–0.995]; wave-II deaths [RR: 0.733; 95% CI: 0.562–0.956]). Similarly, a negative association was observed between COVID-19 deaths and high blood glucose (total waves [RR: 0.963; 95% CI: 0.942–0.984]).

## Discussion

The COVID-19 caseload in the first and the second wave contrast themselves. The gradual increase and decrease during the first wave could be attributed to the early nationwide lockdown and its strict implementation, extensive testing, and other prevention and control strategies. However, the sudden peak and the steeper decline in the second wave point to the rapid spread of the highly transmissible Delta variant across India and the resultant natural immunity. The significant disparity observed in COVID-19 cases and deaths across the different Indian states is likely influenced by the varying physio-socio-economic-demographic factors in different parts of the country. A comprehensive analysis of the key environmental, demographic, socio-economic, and health variables shows wealth index, literacy rate, other workers’ rate, health services, tobacco use in women, alcohol consumption in men, obesity/overweight in women, anemia in women, and rainfall to be positively associated with COVID-19 cases/deaths in India.

Wealth index and literacy rates are important indicators of COVID-19 epidemiology in this analysis. At the district level in India, a positive correlation (r = 0.65) was observed between literacy rate and wealth score^26^. In India, the states with higher literacy rates have a relatively higher wealth index^27,28^. Worldwide, several studies have reported a negative association between education/literacy rates and COVID-19 mortality rates^29–31^. Literate people tend to seek diagnosis and treatment relatively earlier than people without formal education, as they are more aware of the signs and symptoms related to COVID-19^32^. RT-PCR of nasopharyngeal samples is the most widely used COVID-19 detection technique in India, and the COVID-19 test positivity decreases with delayed testing^33^. In addition to reducing the true caseloads, false negative results also adversely affect contact tracing and other preventive and control measures. Wealth and literacy rates in a community greatly influence health-seeking behavior^34^; this could possibly explain why the Indian states with higher wealth index and literacy rates experienced relatively high caseloads and deaths.

Even though NBRM shows a decreased risk for COVID-19 with an increase in blood glucose levels, the spatial exploratory regression analysis shows a strong overlap between elevated blood pressure/sugar levels and COVID-19 cases. NCDs are well-established risk factors of COVID-19 and are associated with increased severity of the disease^35,36^. Diabetes and hypertension are strongly correlated with higher socio-demographic index^37^. In a wealthy state like Kerala, which is a COVID-19 hotspot, most districts have 30–42% of people with elevated blood sugar levels (> 140 mg/dl). In contrast, in almost all the districts in states like Rajasthan, Bihar, and Jharkhand, where the wealth index and COVID-19 cases are relatively lower, less than 20% of the people have blood sugar levels > 140 mg/dl.

Overweight/obesity in women was also significantly associated with COVID-19 deaths in wave-I. A recent study has reported obesity to have a significant association with increased COVID-19 severity and mortality in India^38^. Obesity is associated with hypertension, cardiovascular disease, and type 2 diabetes—well-documented comorbidities of COVID-19^39^. Anemia has been reported to be a risk factor for COVID-19 cases and deaths^40,41^; NBRM analysis shows a positive association between anemia and COVID-19 cases.

NBRM analysis shows a positive association between other (tertiary) workers’ rate and COVID-19 deaths (wave-I). In India, there is a huge regional disparity in employment, especially in the industrial sector. Tamil Nadu ranks first in employment generation, while Kerala and Karnataka, the other southern states, rank among the top 10^42^. The relatively higher literacy rate and wealth index in these southern states might explain the increased tertiary employment in these states. The positive association between tertiary workers’ and COVID-19 deaths suggests literacy rate and wealth index of the state to directly and indirectly affect COVID-19 deaths in India. The nature of employment in essential services, where one has to work in close proximity with others, commute to the workplace, and are continuously exposed to new contacts, makes these workers highly susceptible to COVID-19^43^. Another reason for the high positivity rate in these workers could be because of the mandated testing and contact tracing policies in services/industrial sectors^44^.

The household density and COVID-19 cases and deaths overlapped in the spatial analysis. Household density and overcrowded households are significant risk factors for COVID-19 incidence and mortality^45,46^. In India, 37% of households have only a single room. Maharashtra, one of the hotspots, has an average household size of 4.6^27^, and ~ 42% of households have only one room to dwell in^47^. To reduce the airborne transmission (by droplets and aerosols) of COVID-19, the Center for Disease Control and Prevention recommends infected individuals maintain a six-foot distance between themselves and the other household members^48^. It may be difficult or impossible for people to comply with this recommendation when they are in overcrowded houses^46^.

The study shows tobacco smoking in women to be positively associated with COVID-19 cases and deaths. Smoking is a risk factor in increasing COVID-19 complications^49,50^. Compared to non-smokers, smokers have a > 1-time risk of increased COVID-19 severity^51^. In our analysis, a strong positive association was observed between tobacco use in women and COVID-19 wave-I cases/deaths. However, tobacco use in men showed a significantly decreased risk of COVID-19 cases and deaths. Hotspot analysis of tobacco consumption shows the central and eastern states of India to be the tobacco hotspots^52^, and in these states, the COVID-19 cases/deaths are relatively lower. As reported earlier^53^, this study also finds a positive association between alcohol consumption in men and COVID-19 cases. Analysis of NFHS-4 shows the southern states, especially Kerala and Tamil Nadu, to be alcohol hotspots^54^, and these are the states that have reported higher caseloads of COVID-19. The spatial mapping of COVID-19 cases shows districts in Kerala, Tamil Nadu, Telangana, Chhattisgarh, Odisha, and the NE states are following an upward trend with 99% significance; several districts in these states are hotspots of alcohol use in India^54^. Kerala, followed by Maharashtra, and NE states were found to be the major COVID-19 hotspots in this analysis.

Analysis shows the health services to be positively associated with COVID-19 cases and deaths. In this pandemic, among the larger states, Kerala and Maharashtra were reported to have the best population-level health services^55^. Several initiatives taken by these state governments significantly helped reduce the mortality rates, even though the incident case rates were substantially higher. Inexpensive and effective campaigns like “Break the Chain”^56^ by the Kerala State Government^57^, the “My family, my responsibility”^58^ campaign by the Maharashtra State Government, and other similar campaigns disseminated health-related COVID-19 information to the common public. Rigorous contact tracing, testing, and home quarantine, supervised by local health authorities and the police department, helped identify more cases in Kerala^59^. A similar innovative model formulated by the Municipal Corporation of Greater Mumbai helped identify more incident cases in Mumbai and Dharavi, in Maharashtra^60^. The approach of “chasing the virus” with its 4Ts: (i) Tracing, (ii) Tracking, (iii) Testing, and (iv) Treating in high-risk slum clusters also served the same purpose^61^. The significant relationship between the number of tests, cases, and death rates^59^ might explain why these states reported a high number of incident cases across the two waves.

In this study, environmental variables did not play a significant role in COVID-19 epidemiology, which is in line with similar findings from India^62,63^. However, in the NBRM analysis, wind speed is negatively associated with COVID-19 cases and deaths. Multiple studies have reported wind speed to play a key role in COVID-19 transmission^64–66^. As the virus can remain active in the air for several hours, low wind speed makes the virus stay longer and concentrated in the atmosphere, which leads to increased COVID-19 cases in that area^64^. Furthermore, rainfall shows a positive association with COVID-19 (wave-I deaths) in the NBRM analysis. The western slope of the Western Ghats and the NE states are among the regions receiving maximum rainfall from both monsoon seasons in India^67^. The districts along the western coast (Kerala and Maharashtra) and the NE states are COVID-19 hotspots, which could explain why rainfall is positively associated with COVID-19.

Globally, exposure to ambient air pollution, especially PM_2.5_, is widely reported as a significant risk factor in COVID-19 incidence and severity^68^. A few Indian studies have also reported parallel findings linking COVID-19 and PM_2.5_^62,69^. We observed a relatively high PM_2.5_ (> 76 μg/m^3^) in the highly populated great plains of India, especially in Uttar Pradesh, Bihar, and West Bengal states that lag behind in terms of wealth index^28^, health services^10^, and literacy rates^70^. However, the results of the regression analyses show PM_2.5_ is negatively associated with cases and deaths in waves-I and II. Hesitancy and/or lack of awareness for COVID-19 testing due to lower literacy rates in the Great Plains might explain the lack of association between COVID-19 and PM_2.5_.

As there are no readily available datasets at the micro-administrative (district) level for assessing wealth and health services, the census data (2011) was used for computing these indices, which could be a limitation of the present study. Other limitations relate to the nature of the ecological study, which does not allow conclusions about causality, as it does not guarantee temporal relationship, and does not meet other criteria for causality. Additionally, the study is limited by the lack of temporal estimates between some independent variables (socio-economic-demographic) and the dependent variables.

## Conclusion

The wealth index shows a strong positive RR for COVID-19 cases and deaths. The literacy rate, other workers’ rate, alcohol consumption (men), anemia in women, health services, and rainfall are the other positively associated indicators of COVID-19 morbidity and mortality in India. These associated indicators identified through extensive spatial and statistical analysis might help guide micro-level public health emergencies in India. The space–time trends and mapping of spatial differences of COVID-19 indicators across different districts of India can assist in devising region-specific public health policies.

### Supplementary Information

Supplementary Information 1.

Supplementary Information 2.

Supplementary Information 3.

Supplementary Information 4.

Supplementary Information 5.

Supplementary Information 6.

Supplementary Information 7.

Supplementary Figure 1.

Supplementary Figure 2.

## Data Availability

The raw COVID-19 datasets used for analysis were obtained from public databases: https://data.covid19india.org/ and https://www.mohfw.gov.in/. The district-wise COVID-19 cases, deaths and independent variables are provided in Supplementary Files 2, 3 and 5, respectively.
