# Supplementary material for: Spatio-temporal epidemiology and associated indicators of COVID-19 (wave-I and II) in India
Source: Sci Rep. 2024 Jan 2;14:220. doi: 10.1038/s41598-023-50363-2 (PMC10761923; doi:10.1038/s41598-023-50363-2)
Supplement: Supplementary file 1 — Supplementary Information 1. [file 41598_2023_50363_MOESM1_ESM.docx]

**Wealth Index (WI)**

The wealth index (WI) represents the wealth status of the households, and is determined by the data collected from the latest available HLPCA census book. The indicators used to compute WI are household condition, dwelling rooms, facilities such as drinking water, latrine, drainage, LPG, banking, communication and transport. The district-wise wealth Index (WI) is calculated by the formula (Equation 1).

$WI'=\frac{\sum X_{1+2...n}}{N},$ $WI=\frac{WI'_{i}-WI'_{\min}}{WI'_{\max}-WI'_{\min}}$……………………………… (1)

where, “WI′” is the wealth index of the *i*^th^ district, “X_1_” is the % of good households, “X_2_” is the % of sum of >= 3 dwelling rooms, “X_3_” is the % of drinking water supply within premises, “X_4_” is the % of households having latrine facility within the premises, “X_5_” is the % of households having closed drainage, “X_6_” is the % of LPG use for cooking, “X_7_” is the % of households availing banking services, “X_8_” is the % of households with TV, computer/laptop, telephone/mobile phone and scooter/ car and “N” is the total number of parameter. Then “WI” is the normalised value of WI′, which is used to represent the district-wise wealth status of the households.
