# Supplementary material for: Spatio-temporal epidemiology and associated indicators of COVID-19 (wave-I and II) in India
Source: Sci Rep. 2024 Jan 2;14:220. doi: 10.1038/s41598-023-50363-2 (PMC10761923; doi:10.1038/s41598-023-50363-2)
Supplement: Supplementary file 6 — Supplementary Information 6. [file 41598_2023_50363_MOESM6_ESM.docx]

| **Table S6.1. Descriptive statistics of the total incidence cases and mortality of COVID-19 in Wave I and II** | | | | | |
| --- | --- | --- | --- | --- | --- |
| **Variables** | **Mean** | **SD** | **Min** | **P50** | **Max** |
| Wave 1 Cases | 17201.38 | 43109.32 | 0 | 5881 | 635586 |
| Wave 2 Cases | 34930.77 | 76023.48 | 0 | 12333 | 838951 |
| Total Cases | 52132.15 | 117052.70 | 0 | 19274 | 1439008 |
| Wave 1 Deaths | 246.92 | 845.39 | 0 | 62 | 11210 |
| Wave 2 Deaths | 448.92 | 1120.24 | 0 | 158 | 14236 |
| Total Deaths | 695.84 | 1892.98 | 0 | 224 | 25446 |
| Household Density | 140.93 | 426.87 | 0.21 | 77.06 | 6599.90 |
| Literacy Rate | 62.25 | 10.45 | 28.77 | 61.79 | 88.74 |
| Agricultural Labourer's Rate | 6.40 | 4.95 | 0.00 | 5.10 | 23.86 |
| Household Industries Population Rate | 0.87 | 0.72 | 0.09 | 0.70 | 8.04 |
| Other Workers’ Rate | 12.94 | 7.06 | 2.69 | 11.54 | 52.22 |
| Wealth Index | 0.38 | 0.21 | 0.00 | 0.34 | 0.99 |
| Forest Cover | 25.27 | 25.48 | 0.17 | 16.27 | 92.00 |
| Minimum Temperature | 10.40 | 6.40 | -26.21 | 10.11 | 25.33 |
| Windspeed | 1.12 | 0.43 | 0.46 | 0.96 | 3.00 |
| Actual evapotranspiration | 33.19 | 17.17 | 0.00 | 35.02 | 87.50 |
| Rainfall | 1291.10 | 792.26 | 184.50 | 1074.30 | 7249.94 |
| Particulate Matter 2.5 | 61.91 | 29.40 | 10.04 | 52.35 | 131.30 |
| High Blood Glucose | 13.58 | 4.77 | 5.25 | 12.95 | 33.40 |
| High Blood Pressure | 22.85 | 5.83 | 9.70 | 22.25 | 45.30 |
| Tobacco use (Women) | 11.49 | 11.85 | 0.10 | 7.70 | 70.60 |
| Tobacco use (Men) | 40.94 | 13.93 | 6.80 | 42.90 | 80.60 |
| Alcohol consumption (Women) | 2.59 | 5.75 | 0.00 | 0.50 | 42.80 |
| Alcohol consumption (Men) | 22.16 | 12.49 | 0.10 | 19.10 | 68.40 |
| Overweight/Obese Women | 22.91 | 10.54 | 3.90 | 20.90 | 53.00 |
| Anaemia Women | 55.85 | 12.06 | 14.90 | 57.20 | 93.50 |
| Health Service | 1593.33 | 2407.40 | 0.00 | 893.00 | 27548.00 |

| **Table S6.2. Multicollinearity Analysis** | |
| --- | --- |
| **Variable** | **Variance Inflation Factor (VIF)** |
| Household Density | 3.57 |
| Literacy Rate | 3.05 |
| Agricultural Labourer's Rate | 2.46 |
| Household Industries Population Rate | 1.17 |
| Other Workers Rate | 4.69 |
| Wealth Index | 4.85 |
| Forest | 2.4 |
| Minimum Temperature | 3.5 |
| Windspeed | 2.28 |
| Actual evapotranspiration | 3.16 |
| Rainfall | 2.32 |
| Particulate Matter 2.5 | 3.77 |
| High Blood Glucose | 3.22 |
| High Blood Pressure | 2.5 |
| Tobacco Women | 3.43 |
| Tobacco Men | 5.13 |
| Tobacco Women | 4.08 |
| Alcohol Men | 3.88 |
| Overweight or Obese Women | 4.12 |
| Anaemia Women | 1.71 |
| Health Service Index | 1.74 |

| **Table S6.3. Pairwise correlation between the variables** | | | | | | | | | | | | | | | |  | |  |  | |  | |  | |  | |  |  | |  |  |
| --- | --- | --- | --- | --- | --- | --- | --- | --- | --- | --- | --- | --- | --- | --- | --- | --- | --- | --- | --- | --- | --- | --- | --- | --- | --- | --- | --- | --- | --- | --- | --- |
| **Variables** | **(HouseholdDensity)** | **(LiteracyRate)** | **(AgriculturalLabourersRate)** | **(HouseholdIndustriesWorkersRa)** | **(OtherWorkersRate)** | **(WealthIndex)** | **(Forest)** | **(Min_Temp)** | **(Windspeed)** | **(AET)** | **(Rainfall)** | **(PM_25)** | **(HighBloodGlucose)** | **(HighBloodPressure)** | **(TobaccoWomen)** | | **(TobaccoMen)** | | | **(AlcoholWomen)** | | **(AlcoholMen)** | | **(OverweightorObeseWomen)** | | **(AnaemiaWomen)** | | | **(HealthServices)** | | |
| Household Density | 1.0 |  |  |  |  |  |  |  |  |  |  |  |  |  |  | |  | | |  | |  | |  | |  | | |  | | |
| Literacy Rate | 0.2 | 1.0 |  |  |  |  |  |  |  |  |  |  |  |  |  | |  | | |  | |  | |  | |  | | |  | | |
| Agricultural Labourer's Rate | -0.1 | -0.1 | 1.0 |  |  |  |  |  |  |  |  |  |  |  |  | |  | | |  | |  | |  | |  | | |  | | |
| Household Industries Population Rate | 0.1 | 0.1 | 0.1 | 1.0 |  |  |  |  |  |  |  |  |  |  |  | |  | | |  | |  | |  | |  | | |  | | |
| Other Workers Rate | 0.3 | 0.7 | -0.3 | 0.1 | 1.0 |  |  |  |  |  |  |  |  |  |  | |  | | |  | |  | |  | |  | | |  | | |
| Wealth Index | 0.3 | 0.6 | -0.3 | 0.1 | 0.7 | 1.0 |  |  |  |  |  |  |  |  |  | |  | | |  | |  | |  | |  | | |  | | |
| Forest cover | -0.1 | 0.2 | -0.3 | -0.2 | 0.1 | -0.1 | 1.0 |  |  |  |  |  |  |  |  | |  | | |  | |  | |  | |  | | |  | | |
| Minimum Temperature | 0.1 | 0.3 | 0.4 | 0.2 | 0.2 | 0.0 | -0.1 | 1.0 |  |  |  |  |  |  |  | |  | | |  | |  | |  | |  | | |  | | |
| Windspeed | 0.1 | 0.4 | 0.1 | 0.0 | 0.4 | 0.3 | 0.0 | 0.4 | 1.0 |  |  |  |  |  |  | |  | | |  | |  | |  | |  | | |  | | |
| Actual evapotranspiration | 0.1 | 0.3 | -0.1 | 0.1 | 0.1 | 0.0 | 0.3 | 0.4 | 0.2 | 1.0 |  |  |  |  |  | |  | | |  | |  | |  | |  | | |  | | |
| Rainfall | 0.0 | 0.3 | -0.2 | -0.1 | 0.3 | 0.0 | 0.6 | 0.2 | 0.2 | 0.5 | 1.0 |  |  |  |  | |  | | |  | |  | |  | |  | | |  | | |
| Particulate Matter 2.5 | 0.1 | -0.3 | 0.0 | 0.1 | -0.4 | -0.1 | -0.5 | -0.1 | -0.4 | 0.0 | -0.4 | 1.0 |  |  |  | |  | | |  | |  | |  | |  | | |  | | |
| High Blood Glucose | 0.2 | 0.5 | 0.1 | 0.1 | 0.4 | 0.3 | 0.0 | 0.6 | 0.5 | 0.5 | 0.3 | -0.2 | 1.0 |  |  | |  | | |  | |  | |  | |  | | |  | | |
| High Blood Pressure | 0.1 | 0.4 | 0.0 | 0.1 | 0.4 | 0.4 | 0.2 | 0.1 | 0.3 | 0.3 | 0.3 | -0.3 | 0.4 | 1.0 |  | |  | | |  | |  | |  | |  | | |  | | |
| Tobacco use (Women) | -0.1 | 0.0 | -0.1 | -0.1 | -0.1 | -0.3 | 0.5 | 0.1 | -0.2 | 0.2 | 0.4 | -0.3 | -0.1 | -0.1 | 1.0 | |  | | |  | |  | |  | |  | | |  | | |
| Tobacco use (Men) | -0.2 | -0.4 | -0.1 | -0.2 | -0.5 | -0.6 | 0.3 | -0.2 | -0.4 | 0.0 | 0.1 | 0.1 | -0.4 | -0.5 | 0.7 | | 1.0 | | |  | |  | |  | |  | | |  | | |
| Alcohol consumption (Women) | -0.1 | -0.2 | -0.2 | -0.1 | 0.0 | -0.2 | 0.4 | -0.2 | 0.0 | 0.1 | 0.3 | -0.3 | -0.1 | 0.2 | 0.3 | | 0.3 | | | 1.0 | |  | |  | |  | | |  | | |
| Alcohol consumption (Men) | -0.1 | 0.0 | -0.1 | 0.0 | 0.1 | -0.1 | 0.5 | -0.1 | 0.1 | 0.3 | 0.3 | -0.3 | 0.0 | 0.3 | 0.3 | | 0.2 | | | 0.7 | | 1.0 | |  | |  | | |  | | |
| Overweight or Obese Women | 0.2 | 0.6 | -0.1 | 0.2 | 0.6 | 0.7 | -0.1 | 0.2 | 0.3 | 0.2 | 0.0 | -0.2 | 0.6 | 0.6 | -0.3 | | -0.7 | | | -0.2 | | 0.0 | | 1.0 | |  | | |  | | |
| Anaemia Women | 0.0 | -0.3 | 0.2 | 0.0 | -0.2 | -0.3 | -0.3 | -0.1 | -0.1 | -0.1 | -0.2 | 0.2 | 0.0 | -0.4 | -0.1 | | 0.1 | | | 0.0 | | -0.1 | | -0.2 | | 1.0 | | |  | | |
| Health Services | 0.4 | 0.3 | -0.1 | 0.2 | 0.4 | 0.4 | -0.2 | 0.2 | 0.2 | 0.0 | 0.0 | 0.0 | 0.2 | 0.1 | -0.2 | | -0.3 | | | -0.1 | | -0.1 | | 0.2 | | 0.0 | | | 1.0 | | |

(c)

(a)

(b)

(d)

(f)

(e)

(i)

(h)

(g)

(l)

(k)

(j)

(o)

(n)

(m)

(r)

(q)

(p)

(s)

**Figure S6.1.** Scatter plots of (a) household density, (b) literacy rate, (c) Agricultural laborer’s rate, (d) other worker’s rate, (e) wealth index, (f) household industries worker’s rate, (g) forest cover, (h) minimum temperature, (i) wind speed, (j) AET, (k) rainfall, (l) PM_2.5_, (m) high blood glucose, (n) high blood pressure, (o) tobacco men, (p) alcohol men, (q) overweight/obese women, (r) anemia women, and (s) health service, with total cases (dependent variable) to visually inspect linearity.

(c)

(b)

(a)

(f)

(e)

(d)

(i)

(g)

(h)

(l)

(k)

(j)

(o)

(n)

(m)

(r)

(q)

(p)

(s)

**Figure S6.2.** Scatter plots of (a) household density, (b) literacy rate, (c) Agricultural labourer’s rate, (d) other worker’s rate, (e) wealth index, (f) household industries worker’s rate, (g) forest cover, (h) minimum temperature, (i) wind speed, (j) AET, (k) rainfall, (l) PM_2.5_, (m) high blood glucose, (n) high blood pressure, (o) tobacco men, (p) alcohol men, (q) overweight/obese women, (r) anemia women, and (s) health service, with total deaths (dependent variable) to visually inspect linearity.
