# Supplementary material for: Spatio-temporal epidemiology and associated indicators of COVID-19 (wave-I and II) in India
Source: Sci Rep. 2024 Jan 2;14:220. doi: 10.1038/s41598-023-50363-2 (PMC10761923; doi:10.1038/s41598-023-50363-2)
Supplement: Supplementary file 7 — Supplementary Information 7. [file 41598_2023_50363_MOESM7_ESM.docx]

**Table S7.1.** Risk ratios for factors associated with COVID-19 total cases.

| **Variables** | **Full Model** | | |  | **Reduced Model** | | | |
| --- | --- | --- | --- | --- | --- | --- | --- | --- |
|  | **RR** | **95% CI** | | **P** | **RR** | **95% CI** | | **P** |
| Household Density | 0.919 | 0.816 | 1.035 | 0.166 |  |  |  |  |
| **Literacy Rate** | 1.016 | 1.004 | 1.029 | 0.010 | **1.021** | **1.011** | **1.032** | **0.000** |
| Agricultural Labourers’ Rate | 1.032 | 0.911 | 1.169 | 0.619 |  |  |  |  |
| Other Workers’ Rate | 1.019 | 0.994 | 1.045 | 0.145 |  |  |  |  |
| **Wealth Index** | 3.189 | 1.422 | 7.151 | 0.005 | **3.577** | **2.062** | **6.205** | **0.000** |
| Forest | 1.017 | 0.939 | 1.100 | 0.685 |  |  |  |  |
| Minimum Temperature | 1.010 | 0.988 | 1.032 | 0.382 |  |  |  |  |
| **Windspeed** | 0.762 | 0.580 | 1.000 | 0.050 | **0.779** | **0.609** | **0.995** | **0.046** |
| AET | 1.005 | 0.997 | 1.013 | 0.198 |  |  |  |  |
| Rainfall | 1.000 | 1.000 | 1.000 | 0.602 |  |  |  |  |
| **PM 2.5** | 0.989 | 0.984 | 0.994 | 0.000 | **0.987** | **0.984** | **0.990** | **0.000** |
| High Blood Glucose | 0.983 | 0.955 | 1.012 | 0.241 |  |  |  |  |
| High blood pressure | 1.001 | 0.980 | 1.022 | 0.921 |  |  |  |  |
| **Tobacco Men** | 0.991 | 0.981 | 1.001 | 0.075 | **0.990** | **0.982** | **0.998** | **0.011** |
| **Alcohol Men** | 1.010 | 1.001 | 1.020 | 0.039 | **1.018** | **1.011** | **1.025** | **0.000** |
| Overweight/obesity women | 1.006 | 0.992 | 1.021 | 0.408 |  |  |  |  |
| Anaemia Women | 1.004 | 0.995 | 1.012 | 0.406 |  |  |  |  |
| **Health Services** | 1.005 | 1.000 | 1.009 | 0.053 | **1.005** | **1.001** | **1.009** | **0.021** |

Likelihood-ratio test: Assumption: Reduced model nested within full model, LR chi2(11) = 8.25, Prob > chi2 = 0.6906


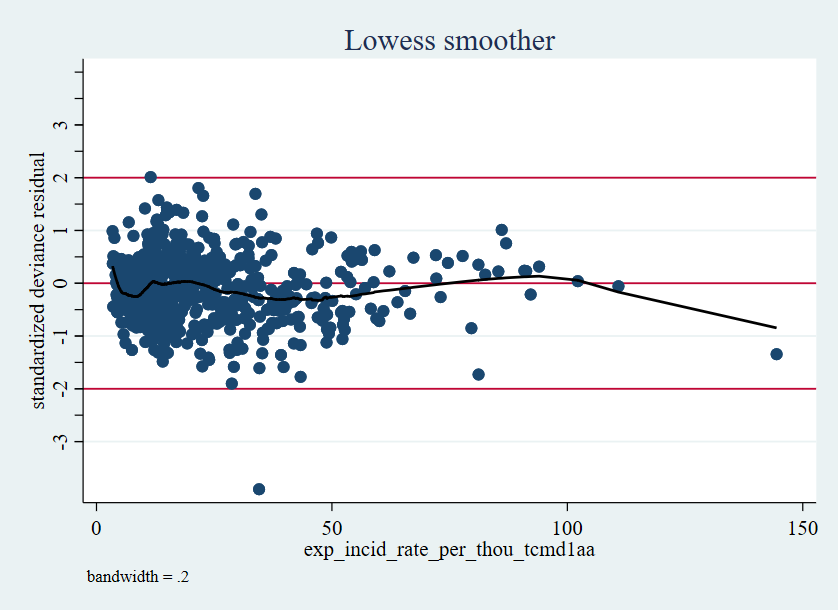


**Figure S7.1.** Residual plot of std deviance residuals against expected number of COVID-19 total cases

**Table S7.2.** Risk ratios for factors associated with COVID-19 wave-I cases.

| **Variables** | **Full Model** | | | | **Reduced Model** | | | |
| --- | --- | --- | --- | --- | --- | --- | --- | --- |
|  | **RR** | **95% CI** | | **p** | **RR** | **95% CI** | | **p** |
| Household Density | 0.992 | 0.877 | 1.122 | 0.900 |  |  |  |  |
| Literacy Rate | 1.005 | 0.993 | 1.017 | 0.450 |  |  |  |  |
| Agricultural Labourers’ Rate | 1.009 | 0.890 | 1.143 | 0.889 |  |  |  |  |
| Other Workers’ Rate | 1.021 | 0.996 | 1.048 | 0.102 | 1.021 | 0.999 | 1.044 | 0.064 |
| **Wealth Index** | 3.395 | 1.508 | 7.647 | 0.003 | **3.854** | **1.886** | **7.878** | **0.000** |
| Forest | 1.023 | 0.945 | 1.106 | 0.579 |  |  |  |  |
| Minimum Temperature | 1.004 | 0.982 | 1.027 | 0.728 |  |  |  |  |
| Windspeed | 0.842 | 0.633 | 1.119 | 0.237 |  |  |  |  |
| AET | 1.005 | 0.997 | 1.012 | 0.241 |  |  |  |  |
| Rainfall | 1.000 | 1.000 | 1.000 | 0.784 |  |  |  |  |
| **PM 2.5** | 0.990 | 0.985 | 0.995 | 0.000 | **0.991** | **0.988** | **0.994** | **0.000** |
| High Blood Glucose | 0.985 | 0.957 | 1.014 | 0.305 |  |  |  |  |
| High blood pressure | 0.990 | 0.969 | 1.012 | 0.387 |  |  |  |  |
| **Tobacco Women** | 1.014 | 1.001 | 1.028 | 0.037 | **1.019** | **1.008** | **1.031** | **0.001** |
| **Tobacco Men** | 0.982 | 0.969 | 0.994 | 0.004 | **0.981** | **0.972** | **0.991** | **0.000** |
| **Alcohol Men** | 1.013 | 1.003 | 1.023 | 0.010 | **1.014** | **1.006** | **1.021** | **0.000** |
| **Anaemia Women** | 1.008 | 1.000 | 1.016 | 0.062 | **1.007** | **1.000** | **1.014** | **0.048** |
| Overweight/obesity women | 1.008 | 0.994 | 1.023 | 0.270 |  |  |  |  |
| **Health Services** | 1.005 | 1.000 | 1.010 | 0.038 | **1.005** | **1.001** | **1.010** | **0.023** |

Likelihood-ratio test: Assumption: Reduced model nested within full model; LR chi2(11) = 8.25, Prob > chi2 = 0.6906


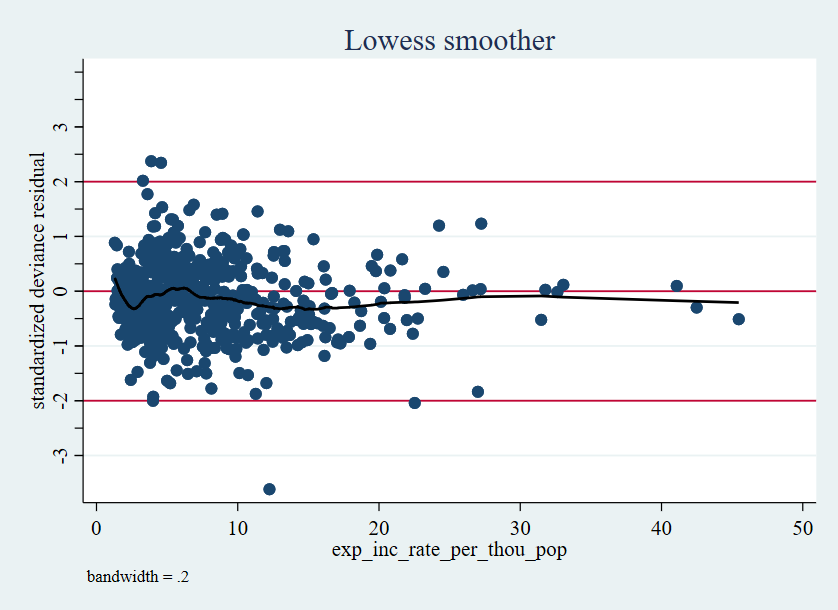


**Figure S7.2.** Residual plot of std deviance residuals against expected number of COVID-19 Wave-I cases

**Table S7.3.** Risk ratios for factors associated with COVID-19 wave II cases.

| **Variables** | **Full Model** | | | | **Reduced Model** | | | |
| --- | --- | --- | --- | --- | --- | --- | --- | --- |
|  | **RR** | **95% CI** | | **p** | **RR** | **95% CI** | | **p** |
| Household Density | 0.901 | 0.800 | 1.015 | 0.087 |  |  |  |  |
| **Literacy Rate** | 1.021 | 1.009 | 1.034 | 0.001 | **1.027** | **1.016** | **1.037** | **0.000** |
| Agricultural Labourers’ Rate | 1.052 | 0.929 | 1.193 | 0.425 |  |  |  |  |
| Other Workers’ Rate | 1.017 | 0.992 | 1.042 | 0.197 |  |  |  |  |
| **Wealth Index** | 3.060 | 1.357 | 6.904 | 0.007 | **3.350** | **1.914** | **5.863** | **0.000** |
| Forest | 1.020 | 0.942 | 1.104 | 0.633 |  |  |  |  |
| Minimum Temperature | 1.007 | 0.984 | 1.030 | 0.559 |  |  |  |  |
| **Windspeed** | 0.766 | 0.582 | 1.010 | 0.059 | **0.771** | **0.600** | **0.992** | **0.043** |
| AET | 1.005 | 0.997 | 1.012 | 0.250 |  |  |  |  |
| Rainfall | 1.000 | 1.000 | 1.000 | 0.431 |  |  |  |  |
| **PM 2.5** | 0.990 | 0.985 | 0.994 | 0.000 | **0.987** | **0.983** | **0.990** | **0.000** |
| High Blood Glucose | 0.984 | 0.956 | 1.013 | 0.288 |  |  |  |  |
| High Blood Pressure | 1.004 | 0.983 | 1.026 | 0.702 |  |  |  |  |
| **Tobacco Men** | 0.990 | 0.980 | 1.001 | 0.069 | **0.990** | **0.982** | **0.998** | **0.017** |
| **Alcohol Men** | 1.009 | 0.999 | 1.019 | 0.067 | **1.017** | **1.010** | **1.024** | **0.000** |
| Overweight Women | 1.004 | 0.989 | 1.019 | 0.615 |  |  |  |  |
| Anaemia Women | 1.000 | 0.992 | 1.009 | 0.937 |  |  |  |  |
| Health Services | 1.005 | 1.000 | 1.010 | 0.054 | 1.004 | 1.000 | 1.009 | 0.051 |

Likelihood-ratio test: Assumption: Reduced model nested within full model; LR chi2(11) = 8.70, Prob > chi2 = 0.6491


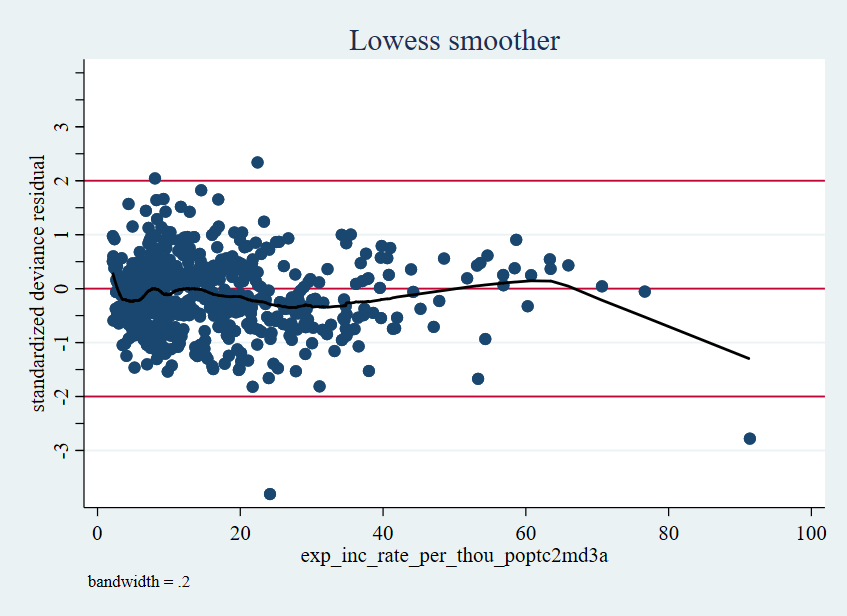


**Figure S7.3.** Residual plot of std deviance residuals against expected number of COVID-19 wave-II cases

**Table S7.4.** Risk ratios for factors associated with COVID-19 total deaths.

| **Variables** | **Full Model** | | | | **Reduced Model** | | | |
| --- | --- | --- | --- | --- | --- | --- | --- | --- |
|  | **RR** | **95% CI** | | **P** | **RR** | **95% CI** | | **P** |
| Household Density | 0.997 | 0.881 | 1.127 | 0.959 |  |  |  |  |
| **Literacy Rate** | 1.033 | 1.020 | 1.047 | 0.000 | **1.040** | **1.028** | **1.052** | **0.000** |
| Agricultural Labourers’ Rate | 1.082 | 0.954 | 1.228 | 0.221 |  |  |  |  |
| Other Workers’ Rate | 1.006 | 0.981 | 1.032 | 0.640 |  |  |  |  |
| **Wealth Index** | 2.695 | 1.179 | 6.162 | 0.019 | **2.477** | **1.361** | **4.506** | **0.003** |
| Forest | 0.971 | 0.897 | 1.051 | 0.467 |  |  |  |  |
| Minimum Temperature | 0.998 | 0.974 | 1.023 | 0.887 |  |  |  |  |
| Windspeed | 0.765 | 0.575 | 1.018 | 0.066 | 0.780 | 0.606 | 1.004 | 0.054 |
| AET | 1.006 | 0.998 | 1.013 | 0.132 |  |  |  |  |
| Rainfall | 1.000 | 1.000 | 1.000 | 0.925 |  |  |  |  |
| **PM 2.5** | 0.989 | 0.984 | 0.994 | 0.000 | **0.991** | **0.987** | **0.994** | **0.000** |
| **High Blood Glucose** | 0.950 | 0.923 | 0.978 | 0.001 | **0.963** | **0.942** | **0.984** | **0.001** |
| High Blood Pressure | 1.014 | 0.992 | 1.036 | 0.209 |  |  |  |  |
| **Tobacco Women** | 1.011 | 0.998 | 1.024 | 0.111 | **1.015** | **1.004** | **1.026** | **0.006** |
| **Tobacco Men** | 0.983 | 0.970 | 0.996 | 0.012 | **0.974** | **0.964** | **0.983** | **0.000** |
| **Alcohol Women** | 0.947 | 0.853 | 1.051 | 0.306 |  |  |  |  |
| Overweight/obesity Women | 1.008 | 0.992 | 1.023 | 0.331 |  |  |  |  |
| Anaemia Women | 1.001 | 0.992 | 1.010 | 0.787 |  |  |  |  |
| **Health Services** | 1.006 | 1.001 | 1.011 | 0.011 | **1.006** | **1.001** | **1.010** | **0.011** |

Likelihood-ratio test: Assumption: Reduced model nested within full model; LR chi2(11) = 8.4, Prob > chi2 = 0.6774


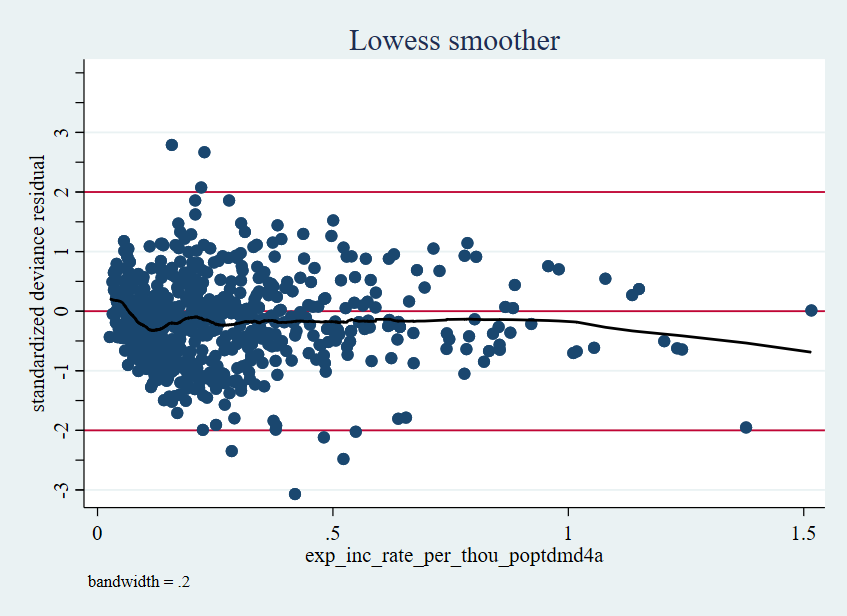


**Figure S7.4.** Residual plot of std deviance residuals against expected number of COVID-19 total deaths

**Table S7.5.** Risk ratios for factors associated with COVID-19 total wave I deaths.

| **Variables** | **Full Model** | | | | **Reduced Model** | | | |
| --- | --- | --- | --- | --- | --- | --- | --- | --- |
|  | **RR** | **95% CI** | | **p** | **RR** | **95% CI** | | **p** |
| Household Density | 1.067 | 0.945 | 1.204 | 0.295 |  |  |  |  |
| **Literacy Rate** | 1.026 | 1.013 | 1.039 | 0.000 | **1.027** | **1.014** | **1.039** | **0.000** |
| Agricultural Labourers’ Rate | 1.115 | 0.984 | 1.264 | 0.087 | 1.125 | 0.995 | 1.273 | 0.060 |
| Household Industries Workers’ Rate | 1.014 | 0.890 | 1.155 | 0.838 |  |  |  |  |
| **Other Workers’ Rate** | 1.026 | 1.000 | 1.053 | 0.047 | **1.026** | **1.001** | **1.051** | **0.042** |
| **Wealth Index** | 3.172 | 1.391 | 7.234 | 0.006 | **3.731** | **1.693** | **8.221** | **0.001** |
| Minimum Temperature | 0.996 | 0.973 | 1.020 | 0.736 | 0.998 | 0.977 | 1.020 | 0.871 |
| Windspeed | 0.934 | 0.704 | 1.240 | 0.638 |  |  |  |  |
| **Rainfall** | 1.000 | 1.000 | 1.000 | 0.072 | **1.000** | **1.000** | **1.000** | **0.049** |
| **PM 2.5** | 0.990 | 0.985 | 0.994 | 0.000 | **0.991** | **0.988** | **0.995** | **0.000** |
| **High Blood Glucose** | 0.930 | 0.904 | 0.957 | 0.000 | **0.933** | **0.907** | **0.960** | **0.000** |
| High Blood Pressure | 1.015 | 0.993 | 1.037 | 0.183 | 1.012 | 0.991 | 1.032 | 0.260 |
| **Tobacco Women** | 1.019 | 1.005 | 1.032 | 0.006 | **1.017** | **1.005** | **1.030** | **0.006** |
| **Tobacco Men** | 0.984 | 0.971 | 0.997 | 0.013 | **0.984** | **0.972** | **0.997** | **0.016** |
| Alcohol Women | 0.961 | 0.861 | 1.072 | 0.477 |  |  |  |  |
| Overweight/obesity Women | 1.019 | 1.004 | 1.035 | 0.012 | **1.020** | **1.005** | **1.036** | **0.007** |
| Anaemia Women | 1.006 | 0.997 | 1.015 | 0.173 | 1.006 | 0.997 | 1.014 | 0.178 |
| **Health Services** | 1.007 | 1.002 | 1.012 | 0.004 | **1.007** | **1.003** | **1.012** | **0.002** |

Likelihood-ratio test: Assumption: Reduced model nested within full model; LR chi2(4) = 2.83, Prob > chi2 = 0.5874


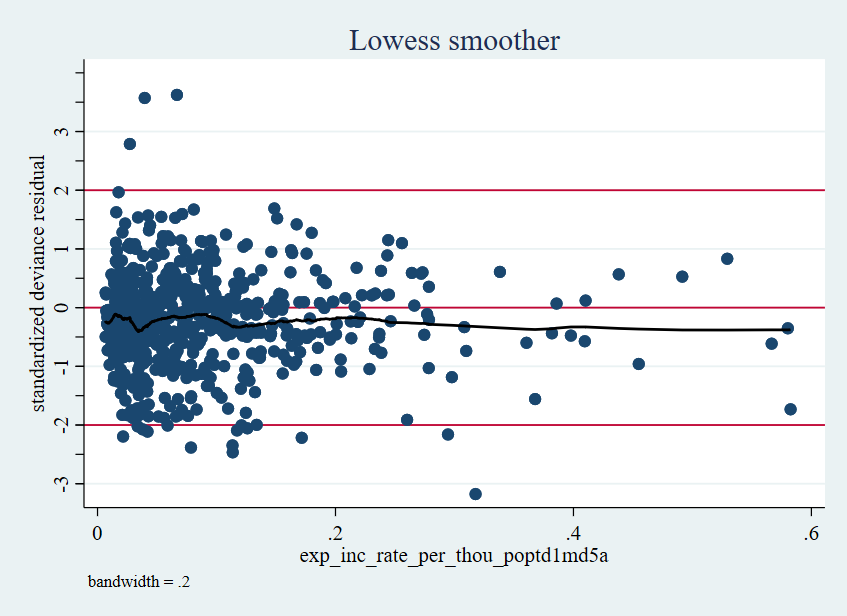


**Figure S7.5.** Residual plot of std deviance residuals against the expected number of COVID-19 wave-I deaths.

**Table S7.6.** Risk ratios for factors associated with COVID-19 total wave II deaths.

| **Variables** | **Full Model** | | | | **Reduced Model** | | | |
| --- | --- | --- | --- | --- | --- | --- | --- | --- |
|  | **RR** | **95% CI** | | **p** | **RR** | **95% CI** | | **p** |
| Household Density | 0.958 | 0.843 | 1.089 | 0.513 |  |  |  |  |
| **Literacy Rate** | 1.037 | 1.023 | 1.051 | 0.000 | **1.037** | **1.024** | **1.051** | **0.000** |
| Agricultural Labourers’ Rate | 1.077 | 0.949 | 1.222 | 0.251 | 1.075 | 0.972 | 1.187 | 0.158 |
| Other Workers’ Rate | 0.997 | 0.972 | 1.023 | 0.811 |  |  |  |  |
| **Wealth Index** | 2.463 | 1.064 | 5.704 | 0.035 | **2.434** | **1.171** | **5.059** | **0.017** |
| Forest | 0.977 | 0.901 | 1.059 | 0.572 |  |  |  |  |
| Minimum Temperature | 1.001 | 0.977 | 1.027 | 0.914 |  |  |  |  |
| **Windspeed** | 0.726 | 0.547 | 0.964 | 0.027 | **0.733** | **0.562** | **0.956** | **0.022** |
| AET | 1.006 | 0.999 | 1.013 | 0.119 | 1.005 | 0.999 | 1.011 | 0.096 |
| Rainfall | 1.000 | 1.000 | 1.000 | 0.562 |  |  |  |  |
| **PM 2.5** | 0.990 | 0.985 | 0.996 | 0.000 | **0.990** | **0.986** | **0.994** | **0.000** |
| **High Blood Glucose** | 0.959 | 0.931 | 0.988 | 0.006 | **0.959** | **0.936** | **0.982** | **0.001** |
| High Blood Pressure | 1.014 | 0.992 | 1.036 | 0.214 | 1.017 | 0.998 | 1.037 | 0.083 |
| Tobacco Women | 1.008 | 0.996 | 1.022 | 0.201 | 1.010 | 0.998 | 1.022 | 0.102 |
| **Tobacco Men** | 0.982 | 0.969 | 0.995 | 0.009 | **0.982** | **0.970** | **0.993** | **0.002** |
| Alcohol Women | 0.947 | 0.853 | 1.052 | 0.309 | 0.942 | 0.862 | 1.029 | 0.186 |
| Overweight/obesity Women | 1.003 | 0.988 | 1.019 | 0.671 |  |  |  |  |
| Anaemia Women | 0.998 | 0.989 | 1.007 | 0.688 |  |  |  |  |
| **Health Services** | 1.006 | 1.001 | 1.011 | 0.017 | **1.005** | **1.001** | **1.010** | **0.025** |

Likelihood-ratio test: Assumption: Reduced model nested within full model; LR chi2(7) = 1.58, Prob > chi2 =0.9793


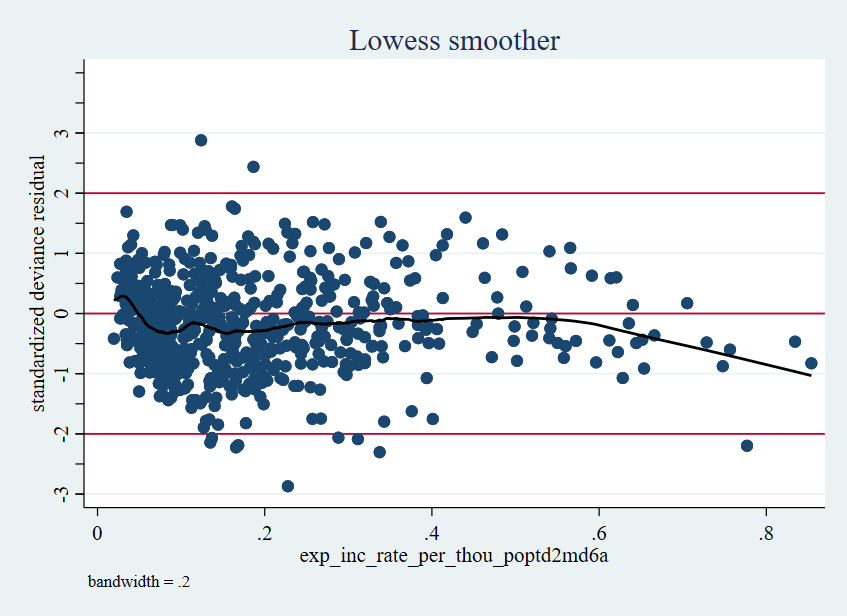


**Figure S7.6.** Residual plot of std deviance residuals against expected number of COVID-19 wave-II deaths.
