# Supplementary material for: Spatio-temporal epidemiology and associated indicators of COVID-19 (wave-I and II) in India
Source: Sci Rep. 2024 Jan 2;14:220. doi: 10.1038/s41598-023-50363-2 (PMC10761923; doi:10.1038/s41598-023-50363-2)
Supplement: Supplementary file 8 — Supplementary Figure 1. [file 41598_2023_50363_MOESM8_ESM.pdf]

Supplementary File 8

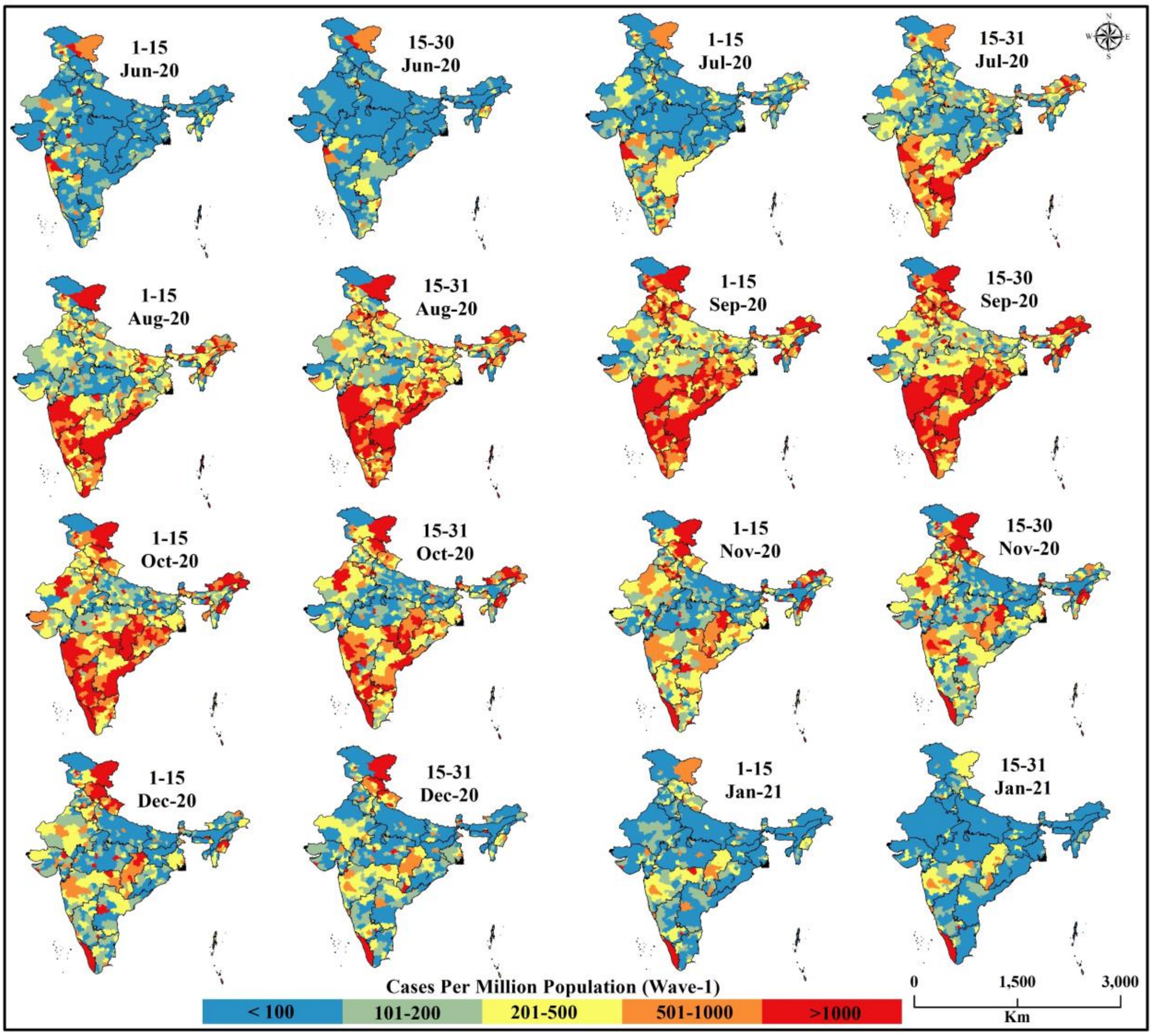

Figure S8.1: District-wise spatio-temporal distribution of 15-day Covid-19 cases per million population during Wave I in India. The maximum cases reported throughout India were from 1-30 September 2020.

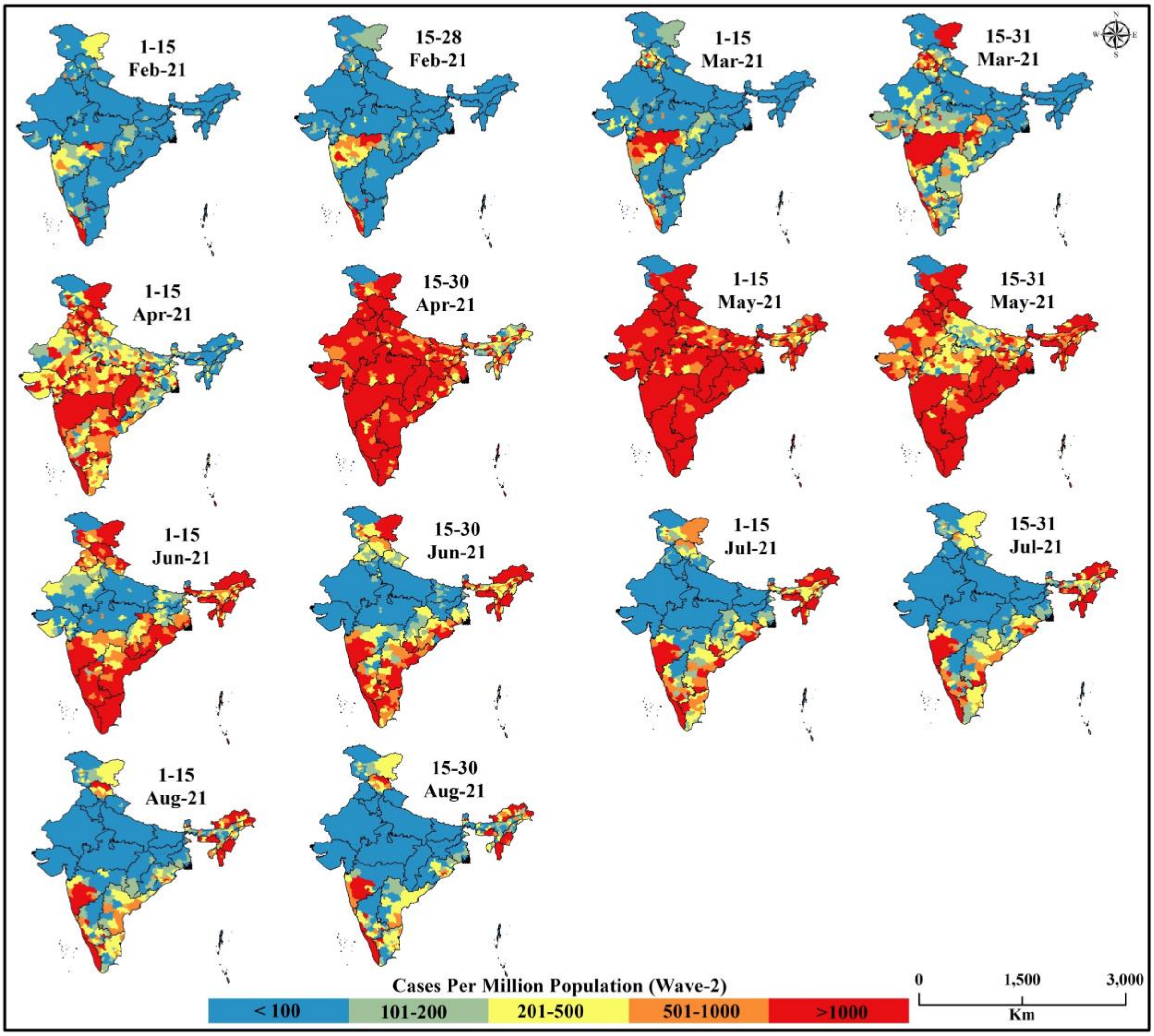

Figure S8.2: District-wise spatio-temporal distribution of 15-day Covid-19 cases per million population during Wave II in India. Except for a few hilly districts, the caseload was maximum during 1-15 May 2021.

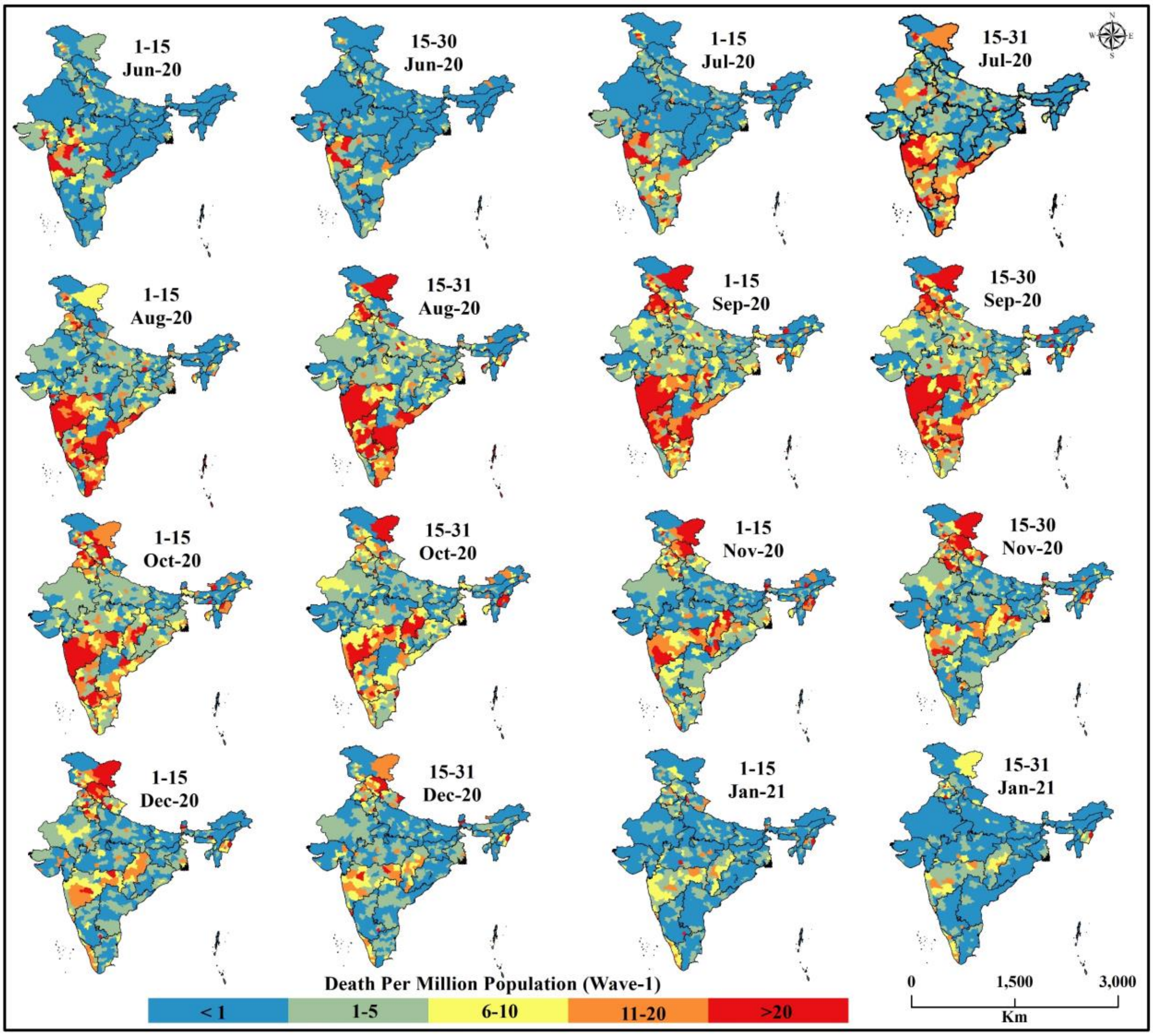

Figure S8.3: District-wise spatio-temporal distribution of 15-day Covid-19 deaths per million population during Wave I in India.

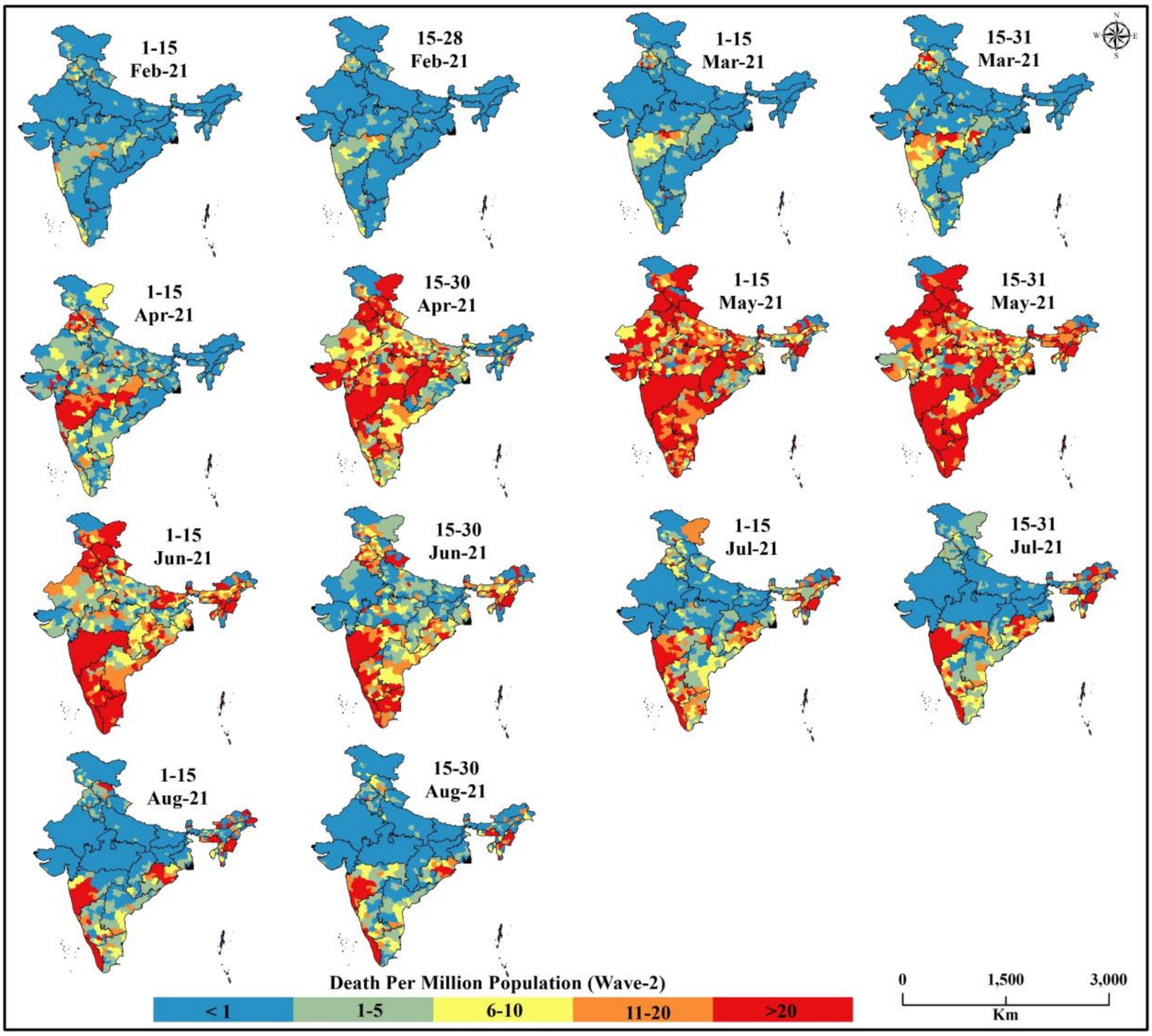

Figure S8.4: District-wise spatio-temporal distribution of 15-day Covid-19 deaths per million population during Wave II in India.
