# Supplementary figures and images for: Spatio-temporal epidemiology and associated indicators of COVID-19 (wave-I and II) in India
Source: Sci Rep. 2024 Jan 2;14:220. doi: 10.1038/s41598-023-50363-2 (PMC10761923; doi:10.1038/s41598-023-50363-2)

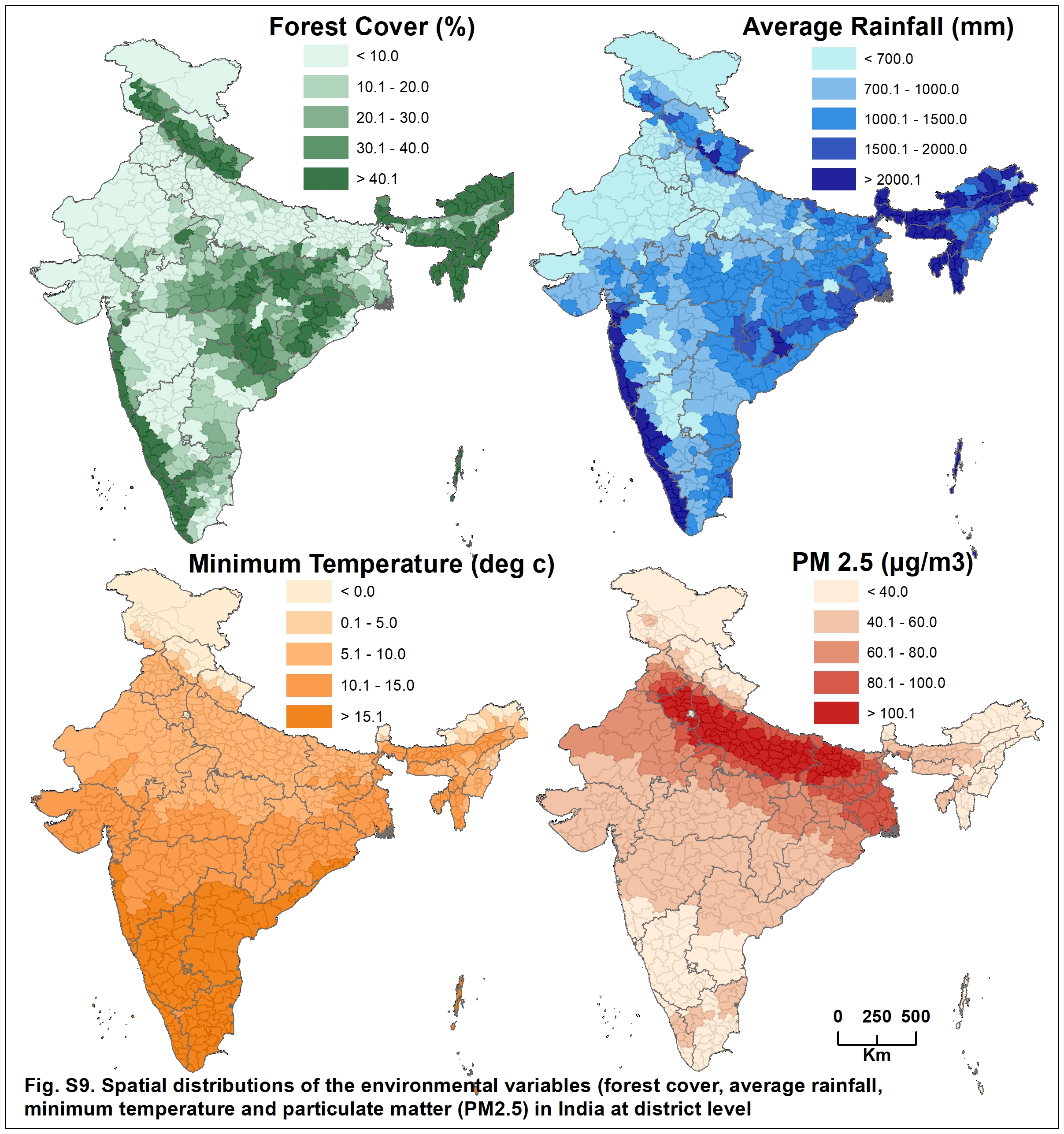

Supplement: Supplementary file 9 — Supplementary Figure 2. [file 41598_2023_50363_MOESM9_ESM.jpg]
